# Multicopy gene family evolution on primate Y chromosomes

**DOI:** 10.1186/s12864-015-2187-8

**Published:** 2016-02-29

**Authors:** Ana-Hermina Ghenu, Benjamin M. Bolker, Don J. Melnick, Ben J. Evans

**Affiliations:** Biology Department, McMaster University, 1280 Main Street West, Hamilton, L8S 4K1 Canada; Department of Mathematics & Statistics, McMaster University, 1280 Main Street West, Hamilton, L8S 4K1 Canada; Department of Ecology, Evolution, and Environmental Biology, Columbia University, 10th Floor Schermerhorn Extension, New York, 10027 USA

**Keywords:** Y chromosome, Ampliconic genes, Gene conversion, Gene duplication, Gene family evolution, Old World Monkeys, Great apes, Genome structure

## Abstract

**Background:**

The primate Y chromosome is distinguished by a lack of inter-chromosomal recombination along most of its length, extensive gene loss, and a prevalence of repetitive elements. A group of genes on the male-specific portion of the Y chromosome known as the “ampliconic genes” are present in multiple copies that are sometimes part of palindromes, and that undergo a form of intra-chromosomal recombination called gene conversion, wherein the nucleotides of one copy are homogenized by those of another. With the aim of further understanding gene family evolution of these genes, we collected nucleotide sequence and gene copy number information for several species of papionin monkey. We then tested for evidence of gene conversion, and developed a novel statistical framework to evaluate alternative models of gene family evolution using our data combined with other information from a human, a chimpanzee, and a rhesus macaque.

**Results:**

Our results (i) recovered evidence for several novel examples of gene conversion in papionin monkeys and indicate that (ii) ampliconic gene families evolve faster than autosomal gene families and than single-copy genes on the Y chromosome and that (iii) Y-linked singleton and autosomal gene families evolved faster in humans and chimps than they do in the other Old World Monkey lineages we studied.

**Conclusions:**

Rapid evolution of ampliconic genes cannot be attributed solely to residence on the Y chromosome, nor to variation between primate lineages in the rate of gene family evolution. Instead other factors, such as natural selection and gene conversion, appear to play a role in driving temporal and genomic evolutionary heterogeneity in primate gene families.

**Electronic supplementary material:**

The online version of this article (doi:10.1186/s12864-015-2187-8) contains supplementary material, which is available to authorized users.

## Background

Gene families are composed of gene copies that were generated by speciation (orthologs) and those that were generated by gene duplication (paralogs). The evolutionary histories of gene families are trimmed by gene loss and intertwined by non-reciprocal recombination (gene conversion), raising the question of whether and how genomic context influences their evolution. One genomic context of interest is the male-specific region of the Y chromosome (msrY) of placental and marsupial (therian) mammals. The origin of this region coincides with the ascendancy of the *SRY* gene as the trigger for the male sex phenotype about 180 million years ago [1–3]. Subsequently, progressively larger portions of the Y and X chromosomes began diverging from one another as large inversions impeded recombination, forming “strata” with differing levels of divergence [4, 5]. During this time, recruitment of alleles with sexually antagonistic function (i.e., alleles that are advantageous to one sex but deleterious to the other) to this region may have been favoured by natural selection [6]. Compared to recombining genomic regions, a lack of recombination rendered the msrY more vulnerable to phenomena that decrease the efficacy of natural selection by Hill-Robertson effects, including Muller’s Ratchet, genetic hitchhiking, and background selection [7, 8]. This had profound consequences over time, including gene loss and the accumulation of repetitive DNA [7]. Today, contemporary eutherian msrYs retain only a small fraction (≈5 *%*) of the genes that were present before divergence from the X chromosome [5]. Male-specific inheritance influenced survival of genes in this region, and surviving genes on the msrY often have male-related functions and expression patterns [9, 10] or are subject to natural selection favoring similar dosage of the proteins they encode in males and females [11]. Examples of gene loss of otherwise conserved Y-linked genes exist, but these are often coupled with translocation to the autosomes or X chromosome [12].

Because the msrY is haploid and paternally inherited, this chromosome is more strongly influenced by genetic drift than the autosomes, which are diploid and biparentally inherited. With equal variance in reproductive success between the sexes, the neutral expectation for the msrY is that its effective population size (*N*_*e*_) is 25 *%* that of the autosomes. This disparity is more pronounced if the variance in reproductive success is higher in males than in females [13, 14], and even more so if the same male individuals monopolize reproduction over multiple generations [15]. Furthermore, in primates, the rate of sequence evolution is faster in males than in females (faster male evolution [16–19]), a factor that could accelerate divergence and deterioration of genes on the msrY.

### Multi-copy ampliconic genes on the msrY

Gene families that include paralogs on the msrY are called ampliconic genes (AGs) [20]. Compared to non-duplicated regions of the Y chromosome that are homologous to the X chromosome, AGs reside in regions that have a higher abundance of genes and pseudogenes but a lower abundance of retrotransposons; the latter observation is possibly a consequence of purifying selection [20–22]. In primates and other mammals, [11], fruit flies [23], and birds [24, 25], intra-chromosomal recombination occurs between AGs. This phenomenon leads to a non-reciprocal transfer where the nucleotide sequence of one duplicate is homogenized by that of another, a process known as gene conversion [26, 27]. On the human msrY, gene conversion occurs frequently – as much as one to four orders of magnitude faster than the nucleotide substitution rate [28–31]. The close proximity on the msrY of direct or inverted (“palindromic”) ampliconic repeats probably facilitates gene conversion [32], although it also occurs less frequently among ampliconic regions that are far apart, including between different chromosome arms [33]. As a result of frequent gene conversion, AG paralogs within either humans or chimpanzees (the tribe Hominini [34]) have higher sequence identity (>99.9 *%*) than orthologous genes [28], even though similarities in copy number and genomic locations across species are consistent with the duplicates having arisen prior to speciation [22].

Gene copy number on the msrY is variable between Old World Primate species [35, 36], and AG copy number polymorphism is also observed within species [37–39], including humans (reviewed in [40]). TSPY copy number variation affects male fertility in humans [41–43] and bulls [44] (but see [45]), suggesting that copy number of this locus is subject to natural selection [20, 46].

### Goals

In this study, our goal is to better understand the evolutionary mechanisms that drive gene family evolution within and among Old World Primate species, with a particular aim of testing whether the nature of gene family evolution of msrY AGs can be distinguished from that of other gene families on the msrY or autosomes. To this end, we collected and estimated phylogenetic relationships among DNA sequences from single copy genes (singletons) and AGs on the msrY of several closely related species of papionin monkey (tribe Papionini), and used a phylogenetic approach to qualitatively assess the frequency of gene conversion in AGs. We then used quantitative PCR (qPCR) to quantify AG copy number variation among and within various species of macaque monkey (genus *Macaca*). Using these data and other information from complete genome sequences from a human, a chimpanzee, and a rhesus macaque (*Macaca mulatta*), we then evaluated the fit of alternative models in which the rate and nature of gene family evolution is allowed to vary among genomic regions and among lineages of Old World Monkeys.

## Results

### Phylogenetic analysis of the primate msrY

Focusing on Old World Primate msrYs, we estimated phylogenetic relationships among msrY sequences from a human, chimp, and rhesus macaque, as well as new sequence data that we collected from several species of papionin monkey. New DNA sequences from four to 14 genes were collected from an olive baboon (*Papio anubis*), a mandrill (*Mandrillus sphinx*), and 15 macaque individuals (genus *Macaca*) from 9 species, including intra-specific information for four macaque species. We inferred the paternal relationships among samples from concatenated singleton genes from the msrY, as well as the phylogenetic relationships within individual AG families including pseudogene sequences obtained from completely sequenced msrY from a human, a chimp, and a rhesus macaque (Additional files 1, 2, 3, 4, 5, 6, 7, 8, 9, 10, 11, 12, 13, 14, 15, 16, 17, 18 and 19). Hereafter we define AGs as any msrY-linked, multi-copy gene family that has been previously demonstrated to have undergone gene conversion in human, chimp, or rhesus macaque; singletons are therefore defined as msrY-linked genes that have not been shown to have undergone gene conversion.

#### Singleton gene tree is consistent with known phylogeny

**Fig. 1:**
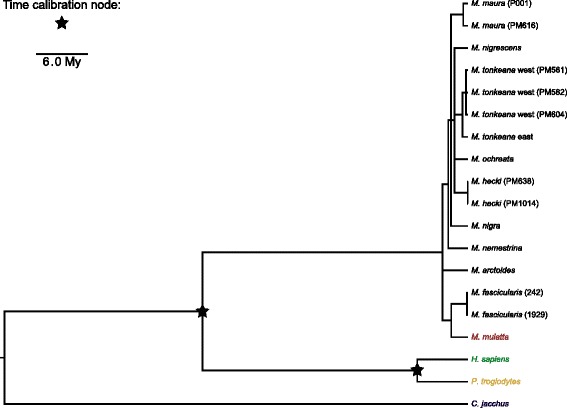
Maximum clade credibility tree of nine concatenated msrY-linked single copy genes. Nodes with less than 95 *%* posterior probability are collapsed. The nodes used for time calibration are labeled with stars. The total alignment length is 6 185 nt. The tree was built with BEAST

Unsurprisingly (because it was constructed from a partially overlapping data set), our estimates of phylogenetic relationships among nine concatenated single copy (singleton) genes on the msrY (*AMELY*, *DBY*, *PRKY*, *SMCY*, *SRY*, *TBL1Y*, *USP9Y*, *UTY*, and *ZFY*; Fig. 1 and Additional file 11) were similar in topology and statistical support to the analysis of [47]. This phylogeny supports, for example, monophyly of the msrY of the Sulawesi macaques and a sister relationship between the msrY of *M. fascicularis* and *M. mulatta*. We added information from two additional samples of *M. maura* and a sample of *M. arctoides*; the phylogenetic placement of these samples was consistent with other studies [48, 49].

#### AG trees support frequent gene conversion in catarrhines

**Fig. 2:**
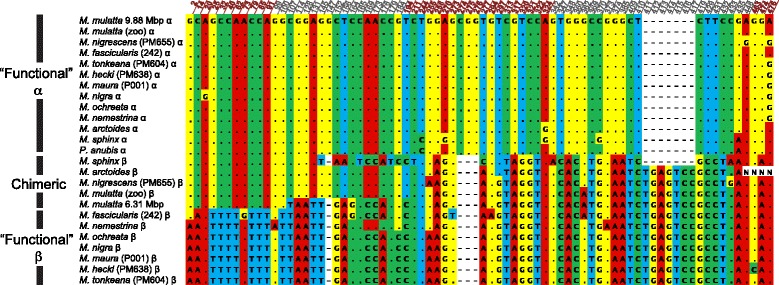
Papionin monkey variable sites in the *TSPY* multiple sequence alignment. Paralogs labeled “ *α*” correspond to sequences of *TSPY2-5*, paralogs labeled “ *β*” correspond to sequences of *TSPY1*, and paralogs labeled “chimeric” have sequences similar to *TSPY2-5* at the 5 ^′^ end and similar to *TSPY1* in the 3 ^′^ end. Dots represent sites that are not different from the rhesus macaque *TSPY2-5* Y-chromosome sequence; letters represent sites that differ. The location (in bp) after primer TSPYex3-5For (Additional file 1: Table S5) are indicated above the alignment; numbers in dark red and gray font show exons and introns, respectively

The gene trees inferred from AG sequences (Additional file 1: Figures S1-S16 and Additional files 12, 13, 14, 15, 16, 17, 18 and 19) provided evidence of gene conversion in terms of (i) the detection of multiple gene sequences (with qPCR or cloning) with lower intraspecific than interspecific sequence divergence, but whose consistent copy number across species suggests ancestral gene duplication, (ii) well supported discordant relationships among putatively orthologous lineages within the gene tree of duplicated genes compared to the gene tree of single copy genes, and (iii) discordant phylogenetic relationships among 5 ^′^ and 3 ^′^ portions of duplicated genes. The first pattern (i) has been noted previously based on data from complete msrY sequences from rhesus, humans, and chimps [21, 22] and is further supported in our analysis by multiple identical or almost identical copies in various macaque individuals identified using qPCR. Specifically, for *HSFY* and *CDY*, sequences from macaques clustered in one clade with two almost identical sequences from the complete msrY of rhesus, but our qPCR results indicated that each of these macaque species carries at least two distinct copies (Additional file 1: Figure S1, Figure S3). Pattern (i) is illustrated by *HSFY* in a baboon (Additional file 1: Figure S3), which has two almost identical copies that cluster together in one clade, whereas other papionins each have two diverged copies that each cluster in different clades, with evolutionary relationships within each clade matching those inferred among singleton genes (Fig. 1). Pattern (ii) is shown by the analysis of *TSPY* (Additional file 1: Figure S5) in that there is strong support for monophyly of all macaques except the Sulawesi macaque *M. nigrescens*, in sharp contrast to the analysis of singleton genes in which all Sulawesi macaques are a clade (Fig. 1). When the 5 ^′^ and 3 ^′^ portions of TSPY are separately analysed, the role of gene conversion becomes apparent because pattern (iii) is shown by at least two independent aspects of this gene tree (Additional file 1: Figure S6). First, *M. arctoides* and one of the rhesus macaques have almost identical sequences at the 5 ^′^ end of *TSPY* but diverged sequences at the 3 ^′^ end (Fig. 2 and Additional file 1: Figure S6). 3 ^′^ end (Fig. 2 and Additional file 1: Figure S6). Second, this same pattern is observed in *M. nigrescens*, presumably due to an independent gene conversion event that altered the 5 ^′^ sequence in the same way in this species but not in other closely related species of Sulawesi macaque (Fig. 2 and Additional file 1: Figure S6). A chimeric sequence was also observed in the reference sequence from the rhesus msrY, suggesting that laboratory artifacts such as PCR chimeras are an unlikely explanation for our observations. These data from *TSPY* could stem from an isolated event early in macaque evolution whose chimeric gene products were detected only in a subset of the species we examined. Alternatively, this could be an example of convergence via multiple independent gene conversion events.

Although not a focus of our study, these phylogenetic analyses supported a close relationship between *DAZ* copies on the msrY and the autosomal gene *DAZL1*, which is consistent with the proposal of [50] that this gene reached the msrY via transposition (Additional file 1: Figure S1) [51]. Similarly, a close relationship between one paralogous msrY lineage of *XKRY* with the autosomal gene *XKR3* is also consistent with an inference of transposition from the msrY to the autosomes (Additional file 1: Figure S7).

### Copy number variation on the macaques’ msrY is low compared to apes

**Fig. 3:**
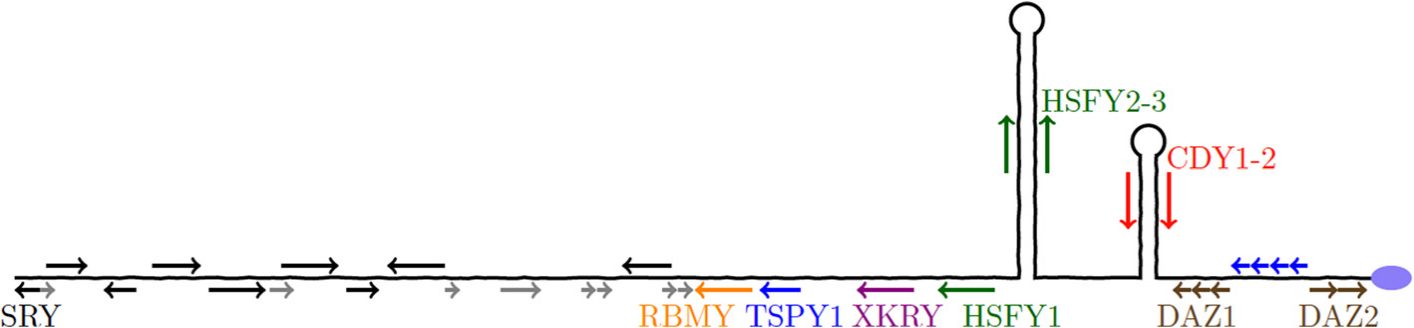
Locations of qPCR-assayed genes on a schematic of the rhesus macaque msrY. Arrows indicate the orientation of protein coding genes. Black arrows indicate singleton loci sequenced in this study, coloured arrows indicate AG loci sequenced in this study, and gray arrows indicate loci not sequenced in this study. Labeled loci indicate genes whose copy numbers were assayed using relative qPCR; *SRY*, *TSPY1*, and *XKRY* were used as reference genes while the others as well as *XKRY* are experimental genes. The blue unlabeled loci correspond to *TSPY2-5*, which was not assayed by qPCR. The multiple arrows for *DAZ1* and *DAZ2* illustrate the exon duplicates within each gene. The two stem-loop structures on the right illustrate palindromes (i.e. inverted repeats) in the ampliconic region and the purple ellipse on the far right illustrates the centromere

We then quantified AG copy number variation in five ampliconic genes (including *RBMY*, *XKRY*, *HSFY*, *CDY*, and *DAZ*; chromosomal locations are shown in Fig. 3) from six to eight species of macaque monkey (*M. nigra*, *M. nigrescens*, *M. hecki*, *M. tonkeana*, *M. maura*, *M. ochreata*, *M. nemestrina*, *M. arctoides*) in seven to 13 individuals using qPCR. We thus assayed copy numbers of all known Old World Primate AGs (including genes found in just one copy in rhesus macaque but in multiple copies in the tribe Hominini), except for *TSPY*. *TSPY* was not analyzed because of high similarity among multiple partially gene converted regions (see above and Fig. 3), which prevented us from developing a robust qPCR assay.

**Fig. 4:**
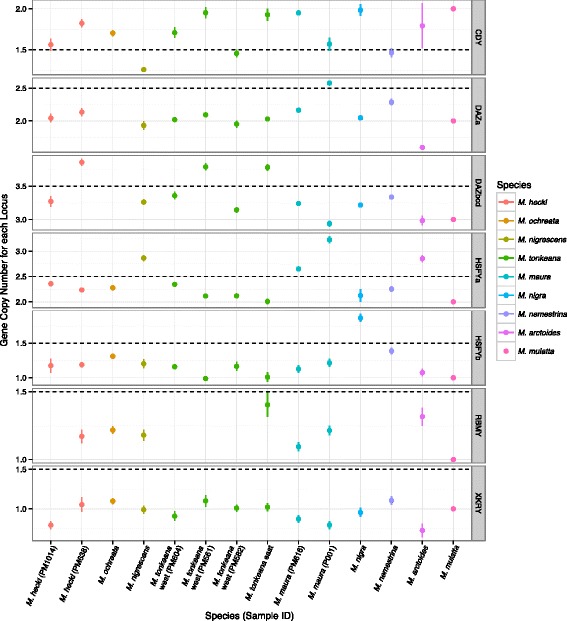
Gene copy numbers for each macaque sample among all seven AG loci assayed by qPCR. The points show the estimated mean copy number and lines depict standard errors, except for the rhesus macaque (*M. mulatta*) where the copy numbers from the Y-chromosome project [22] have been included as a reference. The black dashed lines represent relevant threshold values used for inferring discrete copy numbers from the continuous qPCR data. The lines and points are coloured by species, as indicated in the symbol key on the right

Results of the qPCR analysis are presented in Fig. 4 and Additional file 1: Figures S17–S18. Missing data are either a consequence of failed qPCR assays, as indicated by melt curve analysis (e.g., *RBMY*), or because substitutions in the primer sites prevented the use of a qPCR assay for select species (e.g., the *DAZ*-a assay in *M. ochreata*). We observed considerably less variation in copy number among the macaque species (summed coefficient of variation (CV) among five qPCR assayed AGs =0.396) as compared to that among humans and chimpanzees (summed CV among five AGs =2.99). Assuming constant generation times for all species, a slightly greater amount of time transpired among our sample of macaque species (≈3.1 million generations) compared to that between humans and chimps (≈2.2 million generations), suggesting that AG copy numbers evolve more slowly in macaques than the difference in the coefficients of variation of copy number in each clade would suggest. In fact, because generation time recently became longer in humans, the higher summed CV in the tribe Hominini is even more surprising if rates of gene family evolution were constant across the evolutionary phylogenetic lineages we examined. Nonetheless, as discussed below, the rates of AG family evolution in these lineages are not significantly different.

In general, larger gene families, such as *CDY* and *HSFY*, exhibited more copy number variation among macaque samples (respective CVs =0.201 and 0.148, respectively) than smaller gene families, such as *RBMY* (CV =0.0474) and *XKRY* (CV <10^−6^). This is consistent with the probability of gene duplication being proportional to the number of copies, which is a central feature of the model of gene family evolution used in our analyses discussed below. For *CDY*, one instance of intra-specific copy number polymorphism is suggested for *M. tonkeana*. However, this polymorphism is weakly supported in that the 95 *%* confidence interval spans the copy number threshold. Although present in multiple copies in humans and chimps, *RBMY* and *XKRY* are single-copy in rhesus [22], and we recovered no evidence of multiple copies of these genes in the other macaque species surveyed.

In the reference msrY sequence for rhesus macaque, the *DAZ* gene is present in two copies, and each copy contains tandemly duplicated exons. Based on the rhesus reference msrY, one of our qPCR assays interrogated a triplicated exon in the first *DAZ* gene, *DAZ1* (Fig. 3; qPCR assay *DAZ*-bcd, see Additional file 1), and another assayed a duplicated exon in the second *DAZ* gene, *DAZ2* (Fig. 3; qPCR assay *DAZ*-a, see Additional file 1) [50]. Our results indicate that, similar to the rhesus reference sequence, all of the macaque species we assayed have two copies of *DAZ* (i.e. one copy of *DAZ1* and one of DAZ2). However, within-gene variation in exon number was detected in *DAZ1* in *M. maura* (individual P001 had three copies instead of two), and in *DAZ2* in *M. hecki* and *M. tonkeana* (individuals PM638, PM561, and PM545 had four copies instead of three) (Fig. 4).

### Models of gene family evolution

We developed and evaluated the fit of 6408 models (described below) of gene family evolution to previously published data from chimp, human, and rhesus [20–22, 52] autosomes and msrY, as well as to our new sequence and qPCR data from AG and singleton genes from various species of macaque monkeys. The evolutionary models we considered allowed for unequal rates of gene duplication and deletion (or ‘birth’ and deletion, abbreviated as *BD*) or, alternatively, an equal rate of birth and deletion (*L* model, where *λ*≡*b*=*d*, following [53, 54]).

These models also considered the possibility of two types of rate heterogeneity. The first type, hereafter “lineage heterogeneity”, allows for a different rate of gene family evolution – but the same model of evolution – between the Hominini lineages and the other Old World primate lineages, as previously identified by [52]. The second type, hereafter “gene heterogeneity”, allows for different rates and different evolutionary models of gene family evolution among different classes of gene families. The separate categories considered were the singleton gene families in autosomal DNA, multicopy gene families in autosomal DNA, singleton gene families on the msrY, and AG families on the msrY, and various combinations of these categories. We chose to exclude the *TSPY* gene family (from the msrY) from our analysis because this gene family is a prominent outlier due to its exceptionally high copy number in humans [20]. Inclusion of *TSPY* data in the analyses yielded significantly higher parameter estimates as compared to when this gene family is excluded (data not shown).

Information from completely sequenced Y chromosomes indicates that not all gene families were present on the ancestral Old World Primate msrY, including *TGIF2LY*, PCDH11Y, and *VCY* [3]. *TGIF2LY* and *PCDH11Y* reached the msrY via transposition from the X chromosome in the human lineage [20]; an X-linked homolog of VCY is not present in most mammals and these genes are inferred to have become sex linked during primate evolution [3]. For this reason we decided to also evaluate models that allow a gene family to appear through transposition or to reappear after extinction (abbreviated *I* for ‘innovation,’ following [55]). We explored two types of innovation models. The first set the innovation rate equal to the birth rate (Pr(*X*_*n*+1_=1|*X*_*n*_=0)=*b*; these models are abbreviated *B*=*ID*, if *b*=*i*, or *L*=*I*, if *λ*=*i*). The second, which was explored only in preliminary analyses and then dismissed (see below), estimated the innovation rate (i.e., the transition probability from 0→1 copy, Pr(*X*_*n*+1_=1|*X*_*n*_=0)) independently from the birth/deletion rate(s) (Pr(*X*_*n*+1_=*k*+1|*X*_*n*_=*k*),*k*>0).

**Fig. 5:**
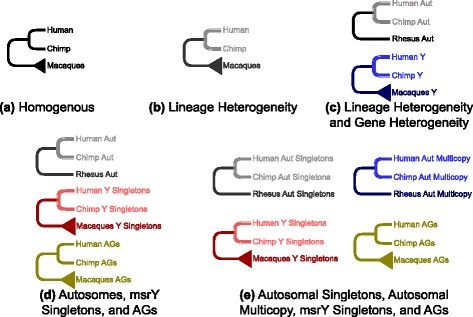
Diagrams of example models fitted to gene copy number data for human, chimp, and macaques. **a** Homogeneous: autosomal genes, msrY-linked singletons, and AGs of all species evolve at the same rate for all lineages. **b** Lineage heterogeneity: Hominini lineage evolves differently from the Old World Primate lineages (shown in gray); autosomal and msrY-linked genes evolve at the same rate(s). **c** Gene heterogeneity and lineage heterogeneity: autosomes (abbreviated “Aut”) and msrY-linked genes (abbreviated “Y”) evolve differently from each other; there is also lineage heterogeneity between Hominini and Old World Primates (illustrated in lighter colours). **d** Autosomal, singleton, and AGs all evolve separately from each other. In this example, there is lineage heterogeneity for the autosomal and singleton genes (illustrated in lighter colours) but lineage homogeneity for the AGs. **e** similar to (d), except that autosomal genes have been separated into singleton (black and gray) and multicopy (navy and blue) gene categories. Triangles at the leaves labeled “Macaques” illustrate that the models were fit to msrY data from the nine macaque species, while leaves labeled “Rhesus” (without triangles) indicate that the models were fit just to the rhesus macaque autosomal data

In summary, Fig. 5 depicts examples of models that we explored, including models in which there is no lineage or gene heterogeneity (Fig. 5a), models in which there is gene and lineage heterogeneity (msrY AGs, msrY singletons, and/or autosomal gene families each have a distinct mode of evolution, with different evolutionary models and/or different parameter values for each gene category), and models in which some of these categories were pooled (e.g., msrY and autosomal singletons pooled, and AGs and autosomal multicopy genes each with a distinct mode of evolution) (Fig. 5b-e). Moreover, for each tree topology depicted in Fig. 5, 8 distinct evolutionary models were considered (namely *L*; *L*=*I*; *BD*; *B*=*ID*; and each of these with or without lineage heterogeneity, see Methods).

We used a threshold method to assign probabilities to each discrete gene copy number from the continuous qPCR data for each sample (see Additional file 1: Methods and Supplementary Information). Our method accommodates uncertainty in copy number inferences based on the qPCR assays, and allows for missing data. Thus we were able to include in our analysis genes for which we had one or more unique sequences but for which we lacked qPCR data. For example, for *AMELY*, we had one unique sequence from each of 13 macaque individuals but we did not have information on copy number variation of this locus. As a conservative measure, this was considered evidence for one or more *AMELY* copies in each of these individuals because we could not exclude the possibility that multiple copies with identical sequences were present in one or more of these species. The data we analyzed from [52] include copy number information from humans, chimps, and the rhesus macaque for 9990 autosomal gene families. However, we lack autosomal gene family data from the eight macaque species whose msrY we investigated with qPCR and sequencing, so these autosomal data were also treated as missing.

#### Models with an independently estimated innovation parameter are not biologically plausible

In our preliminary analyses, in order to check whether inferences from our models were biologically plausible, we additionally evaluated models in which the innovation parameter was independently estimated using the msrY data. We compared the msrY gene maximum *a posteriori* copy numbers predicted at ancestral nodes to information from completely [20–22] or partially sequenced [3, 56] Y chromosomes in primates in order to determine if the gene family ancestral states inferred by models with an independently estimated innovation parameter were consistent with this external information. For example, the existence of a pseudogenized copy of *USP9Y*y in the chimp and functional copies in the human and rhesus Y chromosomes indicates that this gene was present ancestrally in Old World Primates [22]. We found that models with an independently estimated innovation parameter frequently incorrectly inferred the gene family ancestral states. For example, Additional file 1: Figure S19 illustrates that, compared to other models without the innovation parameter, the *LI* and *LI*+lineage heterogeneity models have poor sensitivity in that they fail to identify gene families that were present in the ancestors and instead infer them to be instances of innovation. However, models that allowed innovation were able to correctly identify the autapomorphic human transposition of two singleton gene families (*TGIF2LY* and *PCDH11Y*) that were absent in the ancestor of Old World Primates (true negatives) [20, 22], indicating higher specificity relative to models without an innovation parameter (Additional file 1: Figure S19). But none of the models with innovation were able to correctly identify *VCY* as absent on the most recent common ancestor (MRCA) of Old World Primates, as was proposed by [3, 22].

For these reasons, we excluded these models from our analysis, leaving a total of 6408 for consideration. These models included: all gene categories pooled (8 models); singletons on the autosomes pooled, and with msrY singletons, AGs, and multicopy genes on the autosomes pooled (64 models); autosomes and msrY singletons pooled but AGs separate (64 models); msrY singletons and AGs pooled but autosomes separate (64 models); AGs and autosomes pooled but msrY singletons separate (64 models); autosomal singletons and autosomsal multicopy genes separate but all msrY genes pooled (512 models); autosomal multicopy and AGs separate but autosomal and msrY-linked singleton genes pooled (512 models); autosomal singletons and msrY singletons separate but autosomal multicopy and AGs pooled (512 models); autosomes, msrY singletons, and AGs each separate (512 models); and autosomal singletons, autosomal multicopy genes, msrY singletons, and AGs each separate (4096 models).

#### AG families evolve faster than msrY singletons and autosomes, and msrY singleton may evolve faster than autosomes

**Fig. 6:**
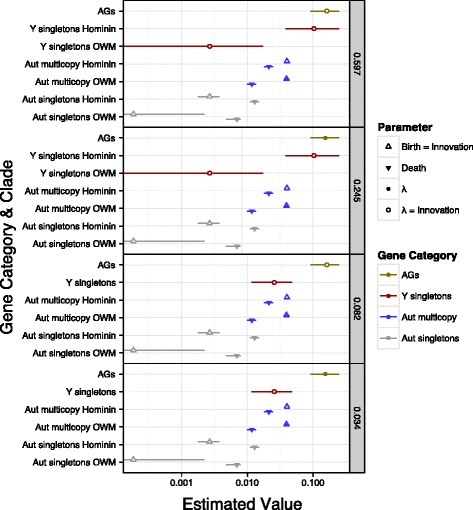
Maximum likelihood estimated (MLE) values for the top 95 *%* cumulative BIC models. Symbols show the MLE and lines indicate univariate 95 *%* confidence intervals. The outlined triangles show the birth=innovation (*b*=*i*) rate estimates and the solid triangles show the deletion (*d*) rate estimates. Estimates for birth and deletion rates (*λ*, *b*, and *d*) are in units of “per gene copy per My,” while estimates of innovation rates (*i*) are in units of “per gene family per My.” The BIC weight of each model is indicated on the far right. On the y-axis, the autosomal gene categories are abbreviated as “Aut,” the singleton categories are labeled “singletons,” and, if a gene category has lineage heterogeneity, its parameters are labeled with either “Hominini” or Old World Monkey (“OWM”). The colours of the symbols highlight gene heterogeneity in all of the models and correspond to different gene categories as indicated in the symbol key on the right

Figure 6 illustrates parameter estimates from the four best models, which together comprise > 95 *%* of the cumulative Bayesian Information Criterion (BIC) weights. These four models share several consistent features. All four support separate evolutionary categories for autosomal singletons, autosomal multicopy genes, msrY-linked singletons, and AGs. Three out of four models, corresponding to 87.6 *%* cumulative BIC weights across all 6408 models, support different mechanisms of gene family evolution among autosomal genes, msrY singletons, and AGs. In all four models, the estimated rate for the AGs is significantly higher than all of the rates for the multicopy autosomal genes (≈4−14 fold for *λ*) and for the singleton autosomal genes (≈12−880 fold for *λ*), and have confidence intervals that do not overlap with those of either of the autosomal gene categories. The birth/deletion rate, *λ*, is significantly higher (≈2−560 fold) in msrY-linked singletons than any of the parameter estimates for autosomal singletons, except for msrY singletons in the tribe Hominini. In all four models, the birth/deletion rate, *λ*, for AGs is significantly higher (≈6−60 fold) than *λ* for msrY singletons in the Old World Monkey lineages other than those of the tribe Hominini. Parameter values for msrY singletons and AGs are presented in Additional file 1: Tables S1 and S2, respectively.

Despite the difference in the CV of AG copy number between Hominini and other Old World Primate lineages discussed above, we did not recover significant support for lineage heterogeneity for AGs. However, lineage heterogeneity is significantly supported for both of the autosomal gene categories. The birth/innovation rate is higher than the death rate (≈2−3 fold) in the multicopy autosomal gene families; but this pattern is reversed, with the death rate ≈3−70 fold higher than the birth/innovation rate, for the singleton autosomal gene families. The rates in the Hominini lineage tend to be faster than in the other Old World monkey lineages (≈1.02−14 fold for birth/innovation, ≈1.8 fold for death) for both autosomal gene categories. Lineage heterogeneity is supported for msrY singletons in two out of the top four models, corresponding to 84.2 *%* cumulative BIC weights across all 6408 models.

In two out of the four most preferred models, corresponding to 27.9 *%* cumulative BIC weights across the 6408 models, the preferred model for AGs evolution did not include an innovation parameter. In contrast, a strong preference (> 95 *%* cumulative BIC weights) for models for the msrY singletons with innovation equal to the birth rate is probably explained by the presence of two X-transposed gene families (*PCDH11Y* and *TGIF2LY*) within this category.

#### Gene family evolution is best explained by a single model for msrY singletons but not AGs

The analysis of autosomes, singletons, and AGs discussed above universally favors models in which AGs evolve separately from the rest of the genome, including singletons on the msrY. Considering just the AGs independently from the other gene categories (see Additional file 1: Table S2), the top three (*L*=*I*, *L*=*I*+lineage heterogeneity, and *B*=*ID*+lineage heterogeneity) of the total eight models have just 61.1 *%* of the cumulative BIC weights, suggesting that there was little power to distinguish between different models of gene family evolution. For a given parameter or suite of related parameters, estimates across the models tended to be similar within each gene category. For example, over most models, the rate of AG birth/deletion (*λ*) or AG birth and AG deletion tended to be around 0.13 events per million generations, or even higher in human and chimpanzee lineages when lineage heterogeneity is allowed.

When we considered just the msrY singletons independently from the other gene categories (see Additional file 1: Table S1), the *L*=*I*+lineage heterogeneity model was preferred with 77.7 *%* of the BIC weight, and the second best of the eight mechanistic models, *B*=*ID*+lineage heterogeneity, was supported by 21.1 *%* of the BIC weight. When the rates of birth and deletion were allowed to differ, the deletion rate in the Old World monkey lineages other than Homonini was inferred to be nearly zero, suggesting that genes present in the ancestor of these Old World monkeys are also generally still present in macaques.

## Discussion

In order to better understand gene family evolution of duplicated ampliconic genes on the primate msrY, we collected qPCR and sequence data from various species of papionin monkey and we analyzed copy number information and DNA sequences from published autosomal and Y chromosomes. We built gene trees to qualitatively evaluate evidence for gene conversion in new and previously available sequence data, including pseudogenes. We then evaluated alternative scenarios of gene family evolution that either imposed or relaxed assumptions of equal rates of gene copy birth, deletion, and innovation; rate consistency over time (lineage homogeneity); and consistency of the model of evolution across gene families of msrY singletons, msrY AGs, and autosomes (gene homogeneity). We recovered strong evidence of gene conversion in many AGs within the msrY, including several novel examples. In *TSPY*, we recovered evidence for multiple independent partial gene convergence events in the same gene, each of which resulted in a chimeric gene product with the 5 ^′^ and 3 ^′^ ends having originated from different ancestral copies.

We also found that gene families evolve significantly faster in msrY AGs than in autosomes, and generally faster than msrY singletons, or perhaps similarly to the birth/deletion rate of msrY singletons in the tribe Hominini when this rate is allowed to vary among lineages (Fig. 6). These results highlight the distinctive nature of AG family evolution, and suggest that this distinctiveness is not solely a consequence of residence on the msrY (because they evolve differently from singletons on the msrY) or genome-wide variation among evolutionary lineages (because they also evolve differently from autosomal gene families).

Another finding that emerged from our analysis is that the inclusion of an independently estimated innovation parameter resulted in biologically unrealistic estimates of other model parameters. Because relatively few genes have been introduced to the msrY since the diversification of Old World monkeys [22], it is unsurprising that there were insufficient innovation events to inform the innovation rate for msrY genes in the species that we investigated. The inclusion of an independently estimated innovation parameter may therefore prove more useful in studying gene family evolution across a broader phylogenetic scope in primates, or in other clades.

### What determines AG evolution?

Our finding of a higher rate of gene family evolution of msrY AGs compared to autosomal gene families matches population genetic expectations if duplicated copies are mildly deleterious and more likely to be observed as intraspecific polymorphisms or fixed differences between species in genomic regions with a small *N*_*e*_. However, this fails to explain why AGs evolve faster than msrY singletons, because both of these gene categories reside on the msrY. For msrY singletons but not AGs, a deletion event represents extinction of the entire gene family within a species, and a birth event leads to a doubling of gene dosage. Thus changes in singleton copy number presumably have a more substantial biological effect than in AGs. That singleton gene families evolve more slowly than those of AGs suggests that singletons are under tighter dosage constraints, and thus more resistant to variation in copy number [3, 11]. Consistent with this speculation, there are multiple examples (including independent examples from the same gene) of msrY-linked loci being lost after a copy is translocated to the autosomes [12].

We classified AGs based on whether they were observed to have experienced gene conversion in humans, chimps, or macaques; AGs thus each have at least two copies of a gene in at least one of these taxa. However, several lines of evidence argue that the separate categories of singletons and AGs are warranted for reasons beyond their copy number in these three taxa. First, msrY singletons and AGs differ substantially in their expression patterns. Singletons are broadly expressed across tissue types and developmental stages [20], and frequently have a counterpart on the X chromosome that escapes X-inactivation in females [57], resulting in a similar protein stoichiometry across both sexes. AGs, in contrast, have testis-specific expression and no counterpart on the X [11, 20]. That all AGs have testis-specific expression suggests that this specificity arose prior to the amplification of AG copy number [11]. Indeed, at the nucleotide level, genes in the msrY singleton and AG categories evolve at significantly different rates, with the rate ratio of nonsynonymous to synonymous substitutions per site being higher in AGs [22]; this disparity is expected based on the different expression patterns [58–60].

Second, msrY singletons do indeed undergo duplication as evidenced by duplications of the *SRY* gene in European rabbits [61] and rats [62], although if the duplication results in a palindrome that undergoes gene conversion, as is the case in the *SRY* gene in rabbits, the nature of gene family evolution may more closely match that of AGs (see our discussion of gene conversion below). Evolutionary history also partially distinguishes singletons and AGs [3, 20, 38, 63]. In primates, all msrY protein coding singleton genes were either present on the mammalian ancestral X and Y chromosomes prior to divergence, or delivered to the Y chromosome from the X (the so called “X-degenerate” and “X-transposed” gene classes, respectively) [20]. However, only about half of the AGs (*HSFY*, *RBMY*, *TSPY*, *VCY*, and *XKRY*y) evolved in this fashion [3, 20, 51]. Two AG families in humans are of unknown origin (*BPY2* and *PRY*), one (*CDY*) was retrotransposed to the msrY from an autosome prior to the diversification of Eutherian mammals [51, 64], and one (*DAZ*) was transposed from an autosome prior to the diversification of Old World Primates [50, 51]. Of the AGs with ancient ancestry on the msrY [3, 51, 64], four (*CDY*, *RBMY*, *TSPY*, and *XKRY*), experienced a pronounced expansion in copy number in the ancestor of catarrhines, and two (*HSFY* and *XKRY*) were subsequently lost in chimps [21, 51, 65].

And third, there are msrY-linked, multi-copy genes which we did not consider to be AGs. *RPS4Y1* and *RPS4Y2*y, for example, are diverged in protein sequence and have not undergone gene conversion for over 35 million years (My) [66, 67] and were thus considered to each be a msrY singleton. Another unusual pair of genes is *CYorf15A*y and *CYorf15B* (known together as *TXLNGY*y), which code for the msrY-linked paralogs of the 5 ^′^ and 3 ^′^ portions, respectively, of the X-linked gene *TXLNG* [68]. This gene probably split into two singleton genes prior to the divergence of Old World Primates [3]. For our analysis, we followed the annotation of [22] for these genes as each functional msrY singletons.

Thus multiple variables distinguish singletons from AGs beyond gene conversion including their expression patterns, molecular evolution, and aspects of their evolutionary history, and these are not strictly a consequence of copy number. Arguably differences in expression patterns and molecular evolution were present ancestrally and thus potentially causal to the observed differing nature of gene family evolution, rather than being consequences of this difference. That the difference in evolutionary rates between msrY singletons and AGs is not strictly a consequence of differences in copy number is also supported by the inference of faster rates of evolution of AGs compared to multicopy autosomal gene families.

### Lineage heterogeneity in autosomes and msrY singletons

The support of our study for lineage heterogeneity of gene family evolution in autosomes is consistent with the study by [52], from which we obtained the autosomal copy number data. This consistency was recovered even though that study used a different statistical approach, with the likelihood approximated using the maximum *a posteriori* (MAP) ancestral state [69] instead of our approach which calculates the likelihood by summing across all ancestral states. Our findings that the birth rate outpaces the deletion rate in autosomes also agrees with previous findings that included data from other Great Apes [70]. In particular, for some of the preferred models with lineage heterogeneity, the estimated deletion rate of autosomes was much higher in Hominini than the estimate of the deletion rate in the other primate lineages.

In the autosomes, the rate of segmental duplications is slower in orangutans than other Great Apes [71], but higher in gorillas and chimps than in humans [70, 72]. However, in rhesus, although small structural variants are abundant [73], these may be a result of mobile element insertion by retrotransposition, as opposed to the larger scale duplication events that are generally responsible for the expansion of gene families [74]. Heterogeneity in the rate of gene family evolution might also arise if this rate was influenced by the extent of sperm competition, as has been proposed in chimps [21, 36, 37, 56, 75]. Inconsistent with this hypothesis, however, are the observations that macaques also have high sperm competition [76–80] yet we recovered relatively low variation in AG copy number among and within macaque species compared to the Hominini.

Some clues toward a mechanistic explanation for lineage heterogeneity in msrY singletons may be gleaned from the three available completely sequenced Old World Primate Y chromosomes, which are distinguished from one another in chromosomal structure. In terms of nucleotide sequences, the rhesus macaque msrY comprises about half (≈11 megabase pairs) of the euchromatin and about one twentieth of AGs (≈0.5 megabase pairs) compared to humans and chimps [22]. A higher content of inverted repeats and repetitive sequences in the Great Apes may promote chromosome fragility, and increase opportunities for duplication or deletion through non-allelic homologous recombination or microhomology-mediated events [81, 82].

Polymorphism in copy number variation in the autosomes appears to be influenced by demographic changes such as bottlenecks. The Western Chimpanzee, for example, has a high level of polymorphism in duplications and deletions, and also has genomic signatures of a population bottleneck [70]. By comparison, there is also evidence for a dynamic demography, including recent population decline in the western population of the Tonkean macaque [83], but our analyses failed to recover compelling evidence of copy number polymorphism based on a limited sample (three individuals; Fig. 4). This disparity could be attributed to a lack of statistical power of our small data-set. We did, however, discover polymorphism at an exon repeat within a paralog of *DAZ* in this population.

### Gene conversion in primates and other species

In general, gene conversion occurs more rapidly in palindromes on the msrY than among palindromic sequences in the autosomes [29], suggesting that the nature of natural selection on duplicates on the Y chromosome may differ from that on duplicates elsewhere in the genome. Relatively few cases of gene conversion within genes on the Y (or W) chromosomes are known beyond those identified in primates based on the complete Y chromosome sequences of a human, a chimp, and a macaque [23]. Known examples include genes that are also arranged in palindromes, including duplicated *SRY* genes in the European rabbit [61], copies of the *HINTW* and *CHD1W* genes in various birds [24, 25], and several genes in cows [39, 84]. Thus our discovery of several clear examples of gene conversion add to a relatively small list of examples from species whose Y chromosomes have yet to be completely sequenced.

Previous studies have considered the evolution of AGs from a theoretical perspective. Ancillary genes that do not themselves undergo gene conversion could catalyze gene conversion of other duplicated genes; these theoretical genes are called recombination modifiers [85]. Using population genetic parameter estimates from humans, simulations indicate that the fixation rate of msrY-linked recombination modifiers can be faster than that for a neutral variant [86]. Simulations and analytical models that jointly consider the phenomena of gene conversion and gene duplication suggest that gene conversion can promote the persistence of gene duplicates on the msrY by resuscitating copies that have undergone deleterious mutation [87]. In this case, gene conversion is not influenced by the fixation probability of a newly arisen duplicate. The theoretical findings of [87] may be supported by the empirical finding of a longer average lifespan of multi-copy genes on the mammalian msrY compared to single-copy genes [11]. The effects of mildly advantageous mutations on duplicates that undergo gene conversion has also been explored, with the conclusion that gene conversion can increase the rate of adaptive evolution [88]. This study noted that gene conversion can be biased, for example, by favoring GC over AT base pairs, and that this bias becomes important when the rate of gene conversion is high [88], which it is on the Y chromosome [28]. Moreover, there is significant evidence of GC biased gene conversion in macaques [83] and other primates [89–92].

Thus, while gene conversion is uniquely possible in multi-copy gene families, theoretical studies suggest that this phenomenon may promote the persistence of gene families on the msrY. In the background of the msrY, whose evolution is dominated by deleterious mutations and strong linkage effects, the role of gene conversion as a conservative force can lead to greater adaptive evolution in AG families [23]. Overall, gene conversion is a plausible causative factor (in addition to being a consequence) of the distinctive nature of AG family evolution.

### Caveats and future directions

#### qPCR assays

An advantage of studying closely related species is that we were able to use multiple gene copy-specific assays to quantify copy number variation across a protracted period of evolutionary time. We anticipate that our qPCR assays accurately identified copy numbers for orthologs that have high sequence identity to the rhesus AG sequences, based on (i) comprehensive sequencing of qPCR primer sites during the development of our qPCR assays, (ii) the high number of technical replicates per individual assay (*n*=4−36 for the experimental samples and *n*=11−34 for the rhesus reference sample), and (iii) the conservative measures we took to identify and exclude replicates with inconsistent reaction efficiencies. However, a drawback (that is difficult to overcome without complete Y chromosome sequences from each species) is that some paralogs may have gone undetected by our qPCR assays if orthologous data were not available in the rhesus macaque due to deletion in an ancestor of rhesus after divergence from the other macaques we assayed.

However, there are two reasons to suspect that our assays did in fact evaluate most or all of the gene families on the macaque msrY. First, if we assume that this rate is similar to that estimated from the available data, the posterior distribution of AG ancestral copy numbers under each of the models, and the relative probability of each model, we would expect, using model averaging, only 0.613 autapomorphic deletion events along the rhesus lineage among all AG families (or, 0.620 deletion events under the preferred model, *L*=*I*). Thus we do not anticipate major differences between the rhesus macaque and the other macaques we surveyed in gene content on the msrY. Second, in our model gene birth and deletion occur at rates that are proportional to the number of copies. For this reason, even if our qPCR assays did systematically fail to identify AG paralogs because they were deleted in the rhesus lineage, this should not bias our estimate of the per copy birth and/or deletion rates. It would, however, mean that we have less information in our data and therefore the confidence intervals on the parameter estimates are larger than they would be if we had more complete data. Further characterization of inter- and intra-specific variation in copy number of primate AGs will undoubtedly increase our understanding of these inferences, and increase their phylogenetic precision. At this time, however, accurate quantification of copy number variation on the msrY is hampered by the repetitive nature of this genomic region, a dearth of completely sequenced Y chromosomes in primates, and by technical complexities associated with assaying copy numbers of genes that are frequently homogenized by gene conversion.

#### Evolutionary models

Our models made several simplifying assumptions that may poorly reflect the actual biological events that occurred during the evolution of gene families on the msrY. For instance, we assumed independence among gene families even though gene families on the msrY are genetically linked. This assumption was made in order to simplify the likelihood calculation. We also assumed that copy number changes of AG families proceed in a stepwise fashion, as has been previously supported [86]. However, it is conceivable that a few rare events may be responsible for multiple duplications happening at the same time – even among different gene families – for example, via crossing over [32, 33, 35, 93] or chromothripsis [94, 95] of the msrY. We also did not include a role of evolutionary ‘strata’ in msrY gene family evolution. However, because the most recent msrY arose prior to the diversification of our species of interest [22], this seems like a reasonable simplifying assumption for our data.

The data we analyzed did not allow for an independently estimated innovation parameter, although most of the preferred models included an innovation parameter at a rate equal to the birth rate. Within a gene category (e.g., msrY singletons) innovation is theoretically possibly via transposition between the sex chromosomes and autosomes [51], resuscitation of pseudogenes through mutation or gene conversion within a gene or across different gene categories [96], and by transitions between gene categories due to copy number evolution (e.g. msrY singletons becoming AGs). To further explore these possibilities it would be interesting to evaluate the effect of an extinction parameter – the transition probability from 1→0 copies, Pr(*X*_*n*+1_=0|*X*_*n*_=1) – in these models, both with and without this parameter being linked to an innovation parameter.

Another factor not considered by our models is the possibility that epistatic interactions between these genes and genes encoded elsewhere in the genome could influence gene family dynamics in unique ways. In particular, if there are favorable combinations of Y-linked and non-Y-linked alleles across genes whose protein products interact, this could favor the translocation from the autosomes or the X chromosome to the Y chromosome in order to prevent these associations from being lost due to recombination. Support for this possibility has been found in fruit flies in which the same Y chromosome exhibits considerable heterogeneity in fitness in different genetic backgrounds [97], and is associated with differential expression of autosomal genes [98].

## Conclusions

This study found multiple novel examples of gene conversion among AGs on the Old World Primate msrY, including one gene that appears to have undergone multiple independent gene conversion events in different species and with similar recombination margins. These independent events yielded chimerical gene products whose evolutionary histories differ between the 5 ^′^ and 3 ^′^ ends of the affected exon. Using data from qPCR, gene sequences, and completely sequenced msrY of a human, chimpanzee, and rhesus macaque, we also demonstrated that AGs on the msrY evolve significantly faster than msrY singletons and autosomal gene families, and that AGs are perhaps better approximated by an altogether distinct model of evolution than those that best approximate the other gene categories. We speculate that the distinctive nature of msrY AGs is a consequence both of the high frequency of gene conversion and natural selection acting on male-specific function of these genes.

## Methods

### Genomic DNA extraction & sequencing

The origins and genomic DNA (gDNA) extraction of samples used in this study are summarized in [47] with the exception of one rhesus macaque, a baboon, and a mandrill sample which were obtained from the Toronto Zoo. Genetic samples for this project were obtained using methods approved by the Institutional Animal Care and Use Committee (IUCAC) at Columbia University.

Sequencing of *TSPY* and *SRY* loci confirmed species identity as determined by [48, 49] and argued against the possibility of inter-specific contamination of Y-chromosome DNA. AG exons in papionins were amplified and sequenced by polymerase chain reaction (PCR) using primers designed from rhesus macaque Y-chromosome bacterial artificial chromosome sequences with high similarity to human AG exons (Additional file 1: Table S3). For all AG loci, multiple primers for at least two exons and/or at different sites were created whenever possible to minimize the possibility of false negative (failed) amplifications due to divergence of primer sites.

### Phylogenetic estimation

A Y-chromosome phylogeny for 14 male macaques (Additional file 1: Table S4), human, chimpanzee, and marmoset was estimated using concatenated nucleotide sequences from up to nine msrY singletons. This analysis included novel sequences for three macaque samples (one *M. arctoides* and two *M. maura* samples), sequences from a marmoset that were identified using BLAST [99], and several other species from a previous study [47] (GenBank accessions in Additional file 1: Table S6). Primers for single-copy, msrY-linked exons and GenBank accessions for the remaining 11 macaque samples, human, and chimpanzee are listed in [47]. The total alignment length was 6185 bp, and the alignment length after excluding positions with gaps was 6167 bp (Additional file 2). The time-calibrated phylogeny was built in BEAST v1.7.5 [100], assuming mean divergence times of 6 My and 30 My for the ancestor of the tribe Hominini and other Old World Primates [101–103], respectively, with model selection, molecular clock calibration, and other analytical details provided in Additional files 1 and 2.

A similar procedure was used for generating the AG trees. Pseudogenes were identified in the completed human, chimp, and rhesus macaque msrY using the functional gene sequences as BLAST queries. In addition to the two calibration dates listed above, a mean divergence time of 8.5 My was assumed for papionins [102, 104, 105] when a putative functional AG ortholog was identified for either mandrill or baboon. Additional files 1, 3, 4, 5, 6, 7, 8, 9 and 10 provide details of the analyses.

### qPCR

Quantitative PCR was performed in accordance with the minimum information for publication of quantitative real-time PCR experiments (MIQE) guidelines [106, 107]. These data are available upon request. Further details of macaque sample processing, gDNA extractions, qPCR primers, targets, amplification parameters, validation, controls, target stability values of reference genes, and intraassay variability are available in the Supplementary Information, and Additional file 1: Tables S4–S12. Standard curves are shown in Additional file 1: Figures S17–S18. gDNA from rhesus macaque was used as a reference sample and assumed to have the same ampliconic gene copy numbers as the individual sequenced for the rhesus macaque Y-chromosome project [22]. Since ampliconic gene copy numbers are unknown for macaque species other than the rhesus macaque and qPCR primers are specific for the genus *Macaca*, no other controls with known copy numbers were available. Therefore, the remaining 13 male macaque samples representing eight species are all part of the experimental group (Additional file 1: Table S4). Samples with divergent sequences at qPCR primer sites (namely DAZa, see Additional file 1: Figure S9), poor assay specificity as determined by melt curve analysis, or assay efficiencies consistently different from the median assay efficiency were excluded from the analysis for the problematic gene family.

qPCR was used to determined the mean expression in each experimental macaque sample relative to rhesus macaque. The known single-copy Y-linked gene *SRY* was used as a reference to confirm the invariant, single-copy status of *TSPY1* and *XKRY* (Fig. 3) for all of the experimental samples. Then, because *TSPY1* and *XKRY* had satisfactory mean stability (geNorm M values) and coefficients of variation (Additional file 1: Table S11), all three loci were used as reference genes to calculate the relative expression for the remaining six loci. Finally, the relative expression for each experimental sample was rescaled to gene copy number using the copy numbers from the rhesus macaque Y-chromosome project [22]. Additional details on qPCR are provided in Additional file 1: Supplementary Methods.

### Copy number estimation from qPCR and sequence data

In order to evaluate the qPCR copy number data in an evolutionary perspective, we needed to generate estimates of the discrete copy numbers for each gene and sample. We assumed that the estimated copy numbers were Normally distributed with a standard deviation equal to the estimated standard error. We assigned a probability to each copy number integer (>0) by calculating the cumulative probability under the density curve for intervals at (0,1.5,2.5,3.5,…). These probabilities were used as the likelihood for the extant taxa, which allowed us to incorporate the uncertainty from the qPCR estimate into our models.

Similarly, for genes and/or samples for which we did not perform qPCR, we used the number of unique sequences observed as an estimate of the minimum number of gene copies present. All copy numbers smaller than the number of unique sequences observed were assumed to have a likelihood of zero, while copy numbers equal to or greater than the number of unique sequences have a likelihood of one.

### Gene family evolution

A homogeneous time Markov process with an arbitrary finite number of states was used to model gene family evolution along the primate phylogeny. Gene duplication and deletion events were modeled using a continuous-time Poisson process where the probability per unit time of an event is proportional to the number of copies. Models *BD* and *B*=*ID* allowed unequal rates of gene duplication, “birth,” and deletion. Models *L*=*I*, *LI*, and *B*=*ID* had an innovation parameter that describes the probability of a gene family moving from zero copies to one copy. Although an innovation parameter has previously been used to model lateral gene transfer of gene families in Prokaryotes [108], innovation may be of particular importance to msrY-linked gene families since it can be used to describe events such as the acquisition of novel gene families on the msrY from the autosomes, the suppression of recombination in part of the pseudo-autosomal region resulting in novel msrY-linked genes, and the putative resuscitation of an extinct gene family by gene conversion of complementary pseudogenes. In models without the innovation parameter, a copy number of zero is an absorbing state; therefore models without innovation assume that each gene family was present in at least one copy in the MRCA of all taxa, while models with innovation do not make this assumption. Furthermore, models without innovation have a limiting distribution at zero and a quasi-stationary distribution at the largest copy number state; both of these are biologically unreasonable distributions for the ancestral state at the MRCA of any gene. We assumed a generation time of five years for all primates [109, 110].

### Missing values and heterogeneous rates

We did not have complete copy number estimates (or minimum values) for all gene families and all macaque species investigated. Therefore, in order to fit the complete data to a single model, we had to accommodate missing data by assigning a likelihood of 1 at all states for genes and taxa with missing data. We implemented rate heterogeneity among lineages in a way that is analogous to its implementation in CAFE [52].

### Analysis of whole genome and msrY data

We downloaded the autosomal gene family size data from [52] and kept only the data from human, chimp, and rhesus macaque. For computational efficiency, we excluded the zinc finger gene family (Family ID ENSFM00250000000002, Ensembl v.82) that has a copy number of >400; this left a total of 9904 autosomal gene families. We subdivided the autosomal data into “singleton” gene families, which only had copy numbers of 1 or 0 in human, chimp, and rhesus macaque, and “multicopy” gene families, which had copy numbers greater than 1 in at least one of the three taxa. About one third (3864) of the autosomal gene families were assigned to the multicopy category. We supplemented these data with the complete msrY gene family size data from [22]. We also included in the data set all of the macaque species by inputting NAs for the autosomal data and the copy numbers determined as described above for the msrY-linked gene data.

All model fitting was performed in R v3.1.0 [111] using custom functions that were based on the function ace from the R package ape v3.0-6 [112]. These functions are available upon request and will be distributed as an R package.

## Animal ethics statement

Genetic samples for this project were obtained using methods approved by the Institutional Animal Care and Use Committee (IUCAC) at Columbia University.

## Availability of supporting data

The new sequence data supporting the results of this article are available in the GenBank repository [sequence accessions KT953619-KT954002]. The sequence alignments and gene trees supporting the results of this article are available in Additional files 2, 3, 4, 5, 6, 7, 8, 9, 10, 11, 12, 13, 14, 15, 16, 17, 18 and 19. The qPCR data sets supporting the results of this article are available upon request.

## Additional files

Additional file 1
**Supplementary Information.** Supplementary methods, supplementary figures, and supplementary tables. (PDF 9.8 Mb)

Additional file 2
**Nine concatenated msrY-linked singleton genes BEAST input file.** Alignment, calibration nodes, nucleotide substitution model, and specification of priors for tree shown in Fig. 1. (XML 129 kb)

Additional file 3
***CDY*** **BEAST input file.** Alignment, calibration nodes, nucleotide substitution model, and specification of priors for tree shown in Additional file 1: Figure S1. (XML 101 kb)

Additional file 4
***DAZ*** **BEAST input file.** Alignment, calibration nodes, nucleotide substitution model, and specification of priors for tree shown in Additional file 1: Figure S2. (XML 139 kb)

Additional file 5
***HSFY*** **BEAST input file.** Alignment, calibration nodes, nucleotide substitution model, and specification of priors for tree shown in Additional file 1: Figure S3. (XML 207 kb)

Additional file 6
***RBMY*** **BEAST input file.** Alignment, calibration nodes, nucleotide substitution model, and specification of priors for tree shown in Additional file 1: Figure S4. (XML 191 kb)

Additional file 7
***TSPY*** **BEAST input file.** Alignment, calibration nodes, nucleotide substitution model, and specification of priors for tree shown in Additional file 1: Figure S5. (XML 86 kb)

Additional file 8
***TSPY*** **5** ^***′***^ **half BEAST input file.** Alignment, calibration nodes, nucleotide substitution model, and specification of priors for left tree shown in Additional file 1: Figure S6. (XML 54 kb)

Additional file 9
***TSPY*** **3** ^***′***^ **half BEAST input file.** Alignment, calibration nodes, nucleotide substitution model, and specification of priors for right tree shown in Additional file 1: Figure S6. (XML 54 kb)

Additional file 10
***XKRY*** **BEAST input file.** Alignment, calibration nodes, nucleotide substitution model, and specification of priors for tree shown in Additional file 1: Figure S7. (XML 33 kb)

Additional file 11
**Nine concatenated msrY-linked singleton genes maximum clade credibility tree.** Summary of 95 *%* high posterior density interval information for tree shown in Fig. 1. (TREE 14.5 KB)

Additional file 12
***CDY*** **maximum clade credibility tree.** Summary of 95 *%* high posterior density interval information for tree shown in Additional file 1: Figure S1. (TREE 70.9 KB)

Additional file 13
***DAZ*** **maximum clade credibility tree.** Summary of 95 *%* high posterior density interval information for tree shown in Additional file 1: Figure S2. (TREE 48.5 KB)

Additional file 14
***HSFY*** **maximum clade credibility tree.** Summary of 95 *%* high posterior density interval information for tree shown in Additional file 1: Figure S3. (TREE 94.2 KB)

Additional file 15
***RBMY*** **maximum clade credibility tree.** Summary of 95 *%* high posterior density interval information for tree shown in Additional file 1: Figure S4. (TREE 157 KB)

Additional file 16
***TSPY*** **maximum clade credibility tree.** Summary of 95 *%* high posterior density interval information for tree shown in Additional file 1: Figure S5. (TREE 106 KB)

Additional file 17
***TSPY*** **5** ^***′***^ **half maximum clade credibility tree.** Summary of 95 *%* high posterior density interval information for left tree shown in Additional file 1: Figure S6. (TREE 104 KB)

Additional file 18
***TSPY*** **3** ^***′***^ **half maximum clade credibility tree.** Summary of 95 *%* high posterior density interval information for right tree shown in Additional file 1: Figure S6. (TREE 106 KB)

Additional file 19
***XKRY*** **maximum clade credibility tree.** Summary of 95 *%* high posterior density interval information for tree shown in Additional file 1: Figure S7. (TREE 52.4 KB)

## Abbreviations

AG: ampliconic gene
*b*: duplication (or ‘ birth’) rate
BAC: bacterial artificial chromosome
BIC: Bayesian information criterion
CV: coefficient of variation
*d*: deletion rate
gDNA: genomic DNA
*i*: innovation rate
*λ*: A rate of copy number evolution where birth and deletion are equal
MAP: maximum *a posteriori*
MRCA: most recent common ancestor
msrY: male-specific region of the Y chromosome
My: million years
*N*_*e*_: effective population size
PCR: polymerase chain reaction
qPCR: quantitative polymerase chain reaction
