# Supplementary material for: Multicopy gene family evolution on primate Y chromosomes
Source: BMC Genomics. 2016 Feb 29;17:157. doi: 10.1186/s12864-015-2187-8 (PMC4772468; doi:10.1186/s12864-015-2187-8)
Supplement: Additional file 1 — Supplementary Information. Supplementary methods, supplementary figures, and supplementary tables. (PDF 9.8 Mb) [file 12864_2015_2187_MOESM1_ESM.pdf]

Multicopy gene family evolution on primate Y chromosomes – Supplementary Information

Ana-Hermina Ghenu   Benjamin M. Bolker
Don J. Melnick   Ben J. Evans

February 22, 2016

Supplementary Methods

Sample details, gDNA extraction, & sequencing

Sequencing of ampliconic exons was accomplished prior to the publication of the complete rhesus macaque Y-chromosome; therefore bacterial artificial chromosome (BAC) sequences from the rhesus macaque Y-chromosome project were used for primer design (GenBank accessions in Table S3). Rhesus macaque sequences with high similarity to human ampliconic exons were used with Primer3 (Untergasser et al., 2012) to design primers for polymerase chain reaction (PCR). For all genes, except *XKRY* and *RBMY*, primers were initially designed to co-amplify putative paralogs and used for direct sequencing. In order to resolve the heterozygous sites resulting from co-amplification of paralogs, the PCR products from at least three Indonesian macaque samples were cloned and sequenced, then, when possible, paralog-specific primers were designed.

Rhesus macaque, mandril, and baboon whole blood samples were obtained from the Toronto Zoo using a needle draw. Blood samples were frozen to -20°C almost immediately after being drawn, shipped on ice to Hamilton, Ontario, then stored continuously at -80°C until DNA extraction. All other macaque samples were obtained from pet animals in Indonesia as detailed in Evans, Morales, Supriatna, and Melnick (1999); Evans, Supriatna, Andayani, and Melnick (2003); Evans, Supriatna, and Melnick (2001). Whole blood was drawn with a needle and fixed almost immediately by mixing with equal parts buffer containing SDS, EDTA, and Tris, as detailed in Evans et al. (1999). Fixed blood samples were stored at room temperature for one to eight weeks before being frozen, then stored continuously at -80°C until DNA extraction.

gDNA was extracted from whole blood for all samples using a DNeasy Blood and Tissue Kit (Qiagen, cat.#69504) with the spin protocol. A modification was made to the manufacturers protocol for the final step: the incubation was done for five minutes with $20 - 80 \mu$L of either distilled water or Buffer AE (as listed in Table tab:samples). The nucleic acid concentration and purity of each gDNA extraction (listed in Table S4) was determined using a NanoDrop ND-1000 Spectrophotometer and the program ND1000 v3.8.1 (ThermoScientific). No DNase or RNase treatment was performed on the gDNA extracts; nor was a contamination assessment carried out on the gDNA extracts. PCR was performed using FailSafe PCR 2x PreMix D

(Epicenter, cat.#FSP995D), and Taq DNA polymerase (Life Technologies); the annealing temperatures are given in Table S9. Primers were tested using genomic DNA from a male rhesus macaque (positive control) and a female *M. ochreata* (negative control) to confirm Y-linkage. PCR reactions were visualized using agarose gel electrophoresis and ethidium bromide. Cloning was done using the TOPO TA kit (Invitrogen, cat.#K4500-40). Sequencing was done using the BigDye Terminator v3.1 Cycle Sequencing kit (Life Technologies, cat.#4337456). Sequence chromatograms were analyzed using Sequencher v4.7 (Gene Codes Corp., Ann Arbour, MI), primer sequences were trimmed in MacClade v4.08 (Sinauer Associates Inc., Sunderland MA, (Maddison & Maddison, 2005)), and FASTA files were aligned using MUSCLE v3.8 (Edgar, 2004).

Phylogenetic estimation

The total alignment length for the concatenated single-copy, msrY-linked, protein coding genes was 6 185 bp, and the alignment length after excluding positions with gaps was 6 167 bp. jModelTest v2.1.3 (Darriba, Taboada, Doallo, & Posada, 2012) was used to estimate the best nucleotide substitution model among 88 candidate models. The model favoured by BIC was TPM3uf+G. Using this model, BEAST v1.7.5 (Drummond, Suchard, Xie, & Rambaut, 2012) was used to create a time-calibrated tree under the following assumptions: a strict molecular clock, that Hominins and macaques each form monophyletic clades, and that the times, in years, to the most recent common ancestor (TMRCA) have priors of $N(\mu = 6.0 \cdot 10^6, \sigma = 3.0 \cdot 10^5)$ and $N(\mu = 3.0 \cdot 10^7, \sigma = 1.5 \cdot 10^6)$ for Hominins and Catarrhines, respectively. These values were chosen for the TMRCA since the mean values are commonly accepted (McBrearty & Jablonski, 2005; Perelman et al., 2011; Scally et al., 2012) and a standard deviation of 5% of the mean was found to yield more efficient mixing and faster convergence than smaller values. BEASTMC3 v1.7.5 was used to run three chains at default settings. After inspecting the chain for convergence in Tracer v1.5 (Rambaut & Drummond, 2003-2009), a burn-in of 1 million generations was applied, yielding a total chain length of 19 million generations.

A similar procedure was used for generating the AG trees. Pseudogenes were identified in the completed human, chimp, and rhesus macaque msrY by using the functional exon sequences as BLAST queries for all three pair-wise combinations. The longest, non-redundant BLAST hits with $> 80\%$ sequence

identity were used to generate the multiple-sequence alignment. The same assumptions as above were used for building BEAST trees except that a relaxed log Normal molecular clock was used instead of a strict molecular clock, since the alignments include pseudogenes as well as functional genes. In addition, the previously reported speciation times (Delson, 1975, 1980; Perelman et al., 2011) for papionins were used as calibration points (prior $= N(\mu = 8.5 \cdot 10^6, \sigma = 4.25 \cdot 10^5)$) in gene families where baboon and/or mandrill presumed functional ortholog sequences were available. Finally, no assumptions (e.g. of monophyly) were made about the tree topology.

qPCR

The annotation from Hughes, Skaletsky, Brown, et al. (2012) was used to identify functional paralogs for qPCR assay in macaques. qPCR primers were designed according to the recommendations of D'haene, Vandesompele, and Hellemans (2010) using Primer3Plus (Untergasser et al., 2012) and the AG sequence alignments obtained from macaques. To ensure a consistent reaction efficiency across samples, primers were placed at sites with 100% identical sequence for all paralogs targeted and in all species assayed (Figures S8-S16). To ensure primer specificity to the targeted paralog(s), primers were designed in regions that had distinguishing sequences in all macaque species as compared to other functional or pseudogenized paralogs; in particular, each pair of primers was required to have at least two distinguishing substitutions within the first five nucleotides on the 3' end (Figures S8-S16). Any potential secondary structure was excluded using the mfold web server (Zuker, 2003) and primer specificity was screened in silico using Primer3Plus (Untergasser et al., 2012) and Primer-BLAST (Ye et al., 2012). Amplicon lengths and locations on the rhesus macaque Y-chromosome (GenBank accession PRJNA253406) are given in Table S6. Primers were ordered from Sigma-Aldrich with reverse-phase cartridge purification and without any modifications to the sequences listed in Table S9.

The rhesus macaque blood sample obtained from a single male individual at the Toronto Zoo was defined as the control group. We assumed that this individual has the same copy numbers for all AGs and *SRY* as the individual sequenced by Hughes, Skaletsky, Brown, et al. (2012). The 13 other macaque samples were defined as the experimental groups because their AG copy numbers are unknown. gDNA extraction methods and purity are described in subsection "gDNA extraction & sequencing," above.

Primer specificity in males was confirmed as detailed in subsection "gDNA extraction & sequencing," above. Specificity was validated using PCR amplification in rhesus macaque followed by either direct sequencing or digestion with a restriction enzyme specific to the mis-primed product (see Table S6). Restriction enzyme digested PCR products were visualized using 8% acrylamide gel electrophoresis and ethidium bromide. Finally, to ensure that primer specificity is consistent across all samples, a melt curve analysis was performed for all reactions to exclude any non-specific reactions.

Primer annealing temperatures were optimized using an eight-point temperature gradient from $55-65$°C. Primer efficiency and linear dynamic range was determined using an eight-point dilution gradient of rhesus macaque gDNA (Figures S17-S18 and Table S8; slope, y-intercept, and r^2 of calibration curves are given in the figure legends of Figures S17-S18). The rhesus macaque gDNA concentration and quantification cycle (C_q) variation at the limit of detection (LOD) for each assay is given in Table S8.

All qPCR reactions were set-up manually and run using a CFX96 Touch Real-Time PCR Detection System (Bio-Rad Laboratories, cat.#185-5196), 96-well PCR plates (Bio-Rad Laboratories, cat.#MLL-9651), and Microseal 'B Adhesive Seals (Bio-Rad Laboratories, cat.#MSB-1001). To minimize the effects of sample-specific inhibition and differential amplification efficiencies between samples, the gDNA concentrations of the experimental samples were normalized relative to the gDNA concentration of the reference sample as recommended by Fernandez-Jimenez et al. (2011) (Table S4). Preliminary assays were done using 8.077μL iTaq Fast SYBR Green Supermix with ROX (Bio-Rad Laboratories, cat.#172-5100), 1μL of gDNA, 0.323μL of 10μM each forward and reverse primers, and UltraPure DNase/RNase-free distilled water (Life Technologies, cat.#10977-015) to a total reaction volume of 15μL. Cycling conditions were: enzyme activation at 95°C for 60s, then 40 cycles of 95°C for 5s, primer specific annealing temperature for 30s, and 70°C for 30s with plate read, and finally a melt curve from $65-95$°C in 0.5°C increments of 5s. After this reagent was discontinued, reference genes were repeated and further assays were done using 5.00μL SsoFast EvaGreen Supermix (Bio-Rad Laboratories, cat.#172-5202), 1μL of gDNA, for some assays 1μL of 10mg/μL bovine serum albumin (BSA) fraction V (Gibco, cat.#11018-017), assay-specific volumes of 10μM primers, and UltraPure DNase/RNase-free distilled water to a total reaction volume of 10μL. Cycling conditions were: enzyme activation at 98°C for 120s, then 40 cycles of 98°C for 5s and primer specific annealing temperature for 5s with plate read, and finally a melt curve

as above. Assay-specific qPCR reaction details (i.e. annealing temperatures, primer concentrations, and whether BSA was added) are listed in Table S7.

C_q values were determined using the baseline-subtracted regression from the CFX Manager Software v3.0 (Bio-Rad Laboratories, n.d.). Average amplification efficiency per sample was estimated using LinRegPCR v2013.1 (Ruijter et al., 2013; Tuomi, Voorbraak, Jones, & Ruijter, 2010). Reactions with baseline errors, as identified by CFX Manager, or efficiencies greater than/less than 8% from the median assay efficiency of the plate, as identified by LinRegPCR, were considered to be kinetic outliers and excluded from further analysis. Inter-run calibration was performed using CFX Manager and the average assay efficiencies estimated from LinRegPCR (Table S8) were used to calculate the relative quantities. The results for the no template control (NTC) reactions is shown in Table S10.

SRY was chosen as a reference gene because it is msrY-linked, rarely lost in mammals (but see (Wallis, Waters, & Graves, 2008)), and usually single-copy in mammals (but see (Bellott et al., 2014; Geraldes, Rambo, Wing, Ferrand, & Nachman, 2010; Lundrigan & Tucker, 1997)). *SRY* was used as a reference to confirm the invariant, single-copy status of *TSPY1* and *XKRY*. We found that using these three reference genes yielded more consistent results for all assays. Finally, the target stability values for these three genes (Table S11) are below the acceptable values for stably expressed reference genes (M-value<0.5 and the coefficient of variation of normalized reference gene relative quantities, CV<0.5; (Hellemans, Mortier, Paepe, Speleman, & Vandesompele, 2007)).

The normalized relative quantities were calculated in CFX Manager. Technical replicates were performed at the qPCR stage. After exclusion of kinetic outliers, the rhesus macaque reference sample had on average 22.1 (min=11, max=34) technical replicates and the experimental samples had on average 16.4 (min=4, max=36) technical replicates among all assays. The intraassay repeatability was quite good, as shown in Table S12.

Copy number estimation from qPCR and sequence data

Unfortunately, previous studies have found that qPCR gene copy number data from gDNA does not cluster cleanly around discrete gene copy numbers (Aldhous et al., 2010; Fode et al., 2011; Nuytten et al., 2009; Perne et al., 2009; Zhang et al., 2014). For this reason, we chose to use a method that incorporates the estimated uncertainty from the qPCR assay to convert the

continuous copy number data into discrete copy number data.

Gene family evolution

Two models, L and BD, are similar to those implemented in CAFE (DeBie, Cristianini, Demuth, & Hahn, 2006; Hahn, De Bie, Stajich, Nguyen, & Cristianini, 2005; Han, Thomas, Lugo-Martinez, & Hahn, 2013). To circumvent the previously documented (Csuros & Miklos, 2009) numerical instability of the probability calculation from Bailey (1964), we used matrix exponentiation for all of the models, as implemented by the expm v0.99-1.1 R package (Goulet et al., 2014), which we found to be numerically stable at copy numbers exceeding 100. Because the Markov process does not have a biologically sensible stationary distribution for models without innovation, we assumed for all models that the ancestral state at the MRCA is Poisson distributed and estimated the Poisson characteristic λ value from the observed copy number data at the tips across all gene families. Note that this prior distribution is different from that used by CAFE, but similar to that used by BadiRate (Librado, Vieira, & Rozas, 2012).

For a proposed parameter value(s), the likelihood was calculated using the pruning algorithm (Felsenstein, 1973) and summing over all the possible reconstructions at the root as weighted by the prior probability of each ancestral state at the MRCA (Pagel, 1994). The maximum likelihood estimate and univariate (profile likelihood) confidence intervals for each model were found using optimization methods in R as implemented by mle2 from the package bbmle v1.0.17 (Bolker, 2014). A bounded method, L-BFGS-B, was used for the optimization because parameter values < 0 are nonsensical for all of the models.

Analysis of whole genome and msrY data

We fitted each of the ten evolutionary models to each of the following gene categories: AGs; msrY-linked singletons; autosomes; AGs and singletons; AGs and autosomes; singletons and autosomes; and AGs, singletons, and autosomes. We calculated the maximum likelihood for all possible combinations of the gene categories that encompassed the entire data by summing the log likelihood of the maximum likelihood estimates for both or all three of these individual model fits.

For each full model, the BIC was estimated using the sum of the number of qPCR observations and the number of copy number estimates from both Hughes, Skaletsky, Brown, et al. (2012) and Hahn, Demuth, and Han (2007) as the sample size ($n = 10040$). The BIC weights and normalized probabilities were calculated using the formulas from Wagenmakers and Farrell (2004).

Supplementary References

References

Aldhous, M. C., Abu Bakar, S., Prescott, N. J., Palla, R., Soo, K., Mansfield, J. C., ... Armour, J. A. L. (2010, nov). Measurement methods and accuracy in copy number variation: Failure to replicate associations of beta-defensin copy number with Crohn's disease. *Hum Mol Gen*, *19*(24), 4930-4938.

Bailey, N. T. J. (1964). *The elements of stochastic processes with application to the natural sciences.* New York: John Wiley & Sons.

Bellott, D. W., Hughes, J. F., Skaletsky, H., Brown, L. G., Pyntikova, T., Cho, T.-J., ... Page, D. C. (2014, apr). Mammalian Y chromosomes retain widely expressed dosage-sensitive regulators. *Nature*, *508*(7497), 494-499.

Bhowmick, B. K., Satta, Y., & Takahata, N. (2007, mar). The origin and evolution of human ampliconic gene families and ampliconic structure. *Genome Res*, *17*(4), 441-450.

Bio-Rad Laboratories. (n.d.). *CFX manager software.* Retrieved from http://www.bio-rad.com/en-ca/product/cfx-manager-software (3.0)

Bolker, B. (2014, May). *bbmle: Tools for general maximum likelihood estimation.* Retrieved from http://cran.r-project.org/web/packages/bbmle/index.html (1.0.17)

Cortez, D., Marin, R., Toledo-Flores, D., Froidevaux, L., Liechti, A., Waters, P. D., ... Kaessmann, H. (2014, apr). Origins and functional evolution of Y chromosomes across mammals. *Nature*, *508*(7497), 488-493.

Csuros, M., & Miklos, I. (2009, aug). Streamlining and large ancestral genomes in Archaea inferred with a phylogenetic birth-and-death model. *Mol Biol Evol*, *26*(9), 2087-2095.

Darriba, D., Taboada, G. L., Doallo, R., & Posada, D. (2012). jModelTest 2: more models, new heuristics and parallel computing. *Nat Methods*, *9*(8), 772–772. Retrieved from https://code.google.com/p/jmodeltest2/

DeBie, T., Cristianini, N., Demuth, J., & Hahn, M. (2006). CAFE: a computational tool for the study of gene family evolution. *Bioinformatics*, *22*(10), 1269.

Delson, E. (1975). Evolutionary history of the Cercopithecidae. *Contrib Primatol*, *5*, 167–217.

Delson, E. (1980). The macaques: Studies in ecology, behavior and evolution. In (pp. 10–30). New York, NY: Van Nostrand Reinhold.

D'haene, B., Vandesompele, J., & Hellemans, J. (2010, apr). Accurate and objective copy number profiling using real-time quantitative PCR. *Methods*, *50*(4), 262-270.

Drummond, A. J., Suchard, M. A., Xie, D., & Rambaut, A. (2012). Bayesian phylogenetics with BEAUti and the BEAST 1.7. *Mol Biol Evol*, *29*(8), 1969–1973. Retrieved from http://beast.bio.ed.ac.uk/

Edgar, R. C. (2004). MUSCLE: multiple sequence alignment with high accuracy and high throughput. *Nucleic Acids Res*, *32*(5), 1792–1797. Retrieved from http://www.drive5.com/muscle/

Evans, B. J., Morales, J. C., Supriatna, J., & Melnick, D. J. (1999). Origin of the Sulawesi macaques (Cercopithecidae: *Macaca*) as suggested by mitochondrial DNA phylogeny. *Biol J Linnean Soc*, *66*(4), 539–560.

Evans, B. J., Supriatna, J., Andayani, N., & Melnick, D. J. (2003). Diversification of Sulawesi macaque monkeys: decoupled evolution of mitochondrial and autosomal DNA. *Evolution*, *57*(8), 1931–1946.

Evans, B. J., Supriatna, J., & Melnick, D. J. (2001). Hybridization and population genetics of two macaque species in Sulawesi, Indonesia. *Evolution*, *55*(8), 1686–1702.

Felsenstein, J. (1973, sep). Maximum likelihood and minimum-steps methods for estimating evolutionary trees from data on discrete characters. *Syst Zool*, *22*(3), 240-249.

Fernandez-Jimenez, N., Castellanos-Rubio, A., Plaza-Izurieta, L., Gutierrez, G., Irastorza, I., Castaño, L., ... Bilbao, J. R. (2011, dec). Accuracy in copy number calling by qPCR and PRT: A matter of DNA. *PLOS ONE*, *6*(12), e28910.

Fode, P., Jespersgaard, C., Hardwick, R. J., Bogle, H., Theisen, M., Dodoo, D., ... Hollox, E. J. (2011, feb). Determination of beta-defensin genomic copy number in different populations: A comparison of three methods. *PLOS ONE*, *6*(2), e16768.

Geraldes, A., Rambo, T., Wing, R. A., Ferrand, N., & Nachman, M. W. (2010, Nov). Extensive gene conversion drives the concerted evolution of paralogous copies of the *SRY* gene in european rabbits. *Mol Biol Evol*, *27*(11), 2437-40.

Goulet, V., Dutang, C., Maechler, M., Firth, D., Shapira, M., & Stadel-

mann, M. (2014, February). *expm: Matrix exponential.* Retrieved from http://cran.r-project.org/web/packages/expm/index.html (0.99-1.1)

Hahn, M. W., De Bie, T., Stajich, J. E., Nguyen, C., & Cristianini, N. (2005, aug). Estimating the tempo and mode of gene family evolution from comparative genomic data. *Genome Res*, *15*(8), 1153-1160.

Hahn, M. W., Demuth, J. P., & Han, S. G. (2007, nov). Accelerated rate of gene gain and loss in primates. *Genetics*, *177*(3), 1941-1949.

Han, M. V., Thomas, G. W. C., Lugo-Martinez, J., & Hahn, M. W. (2013, jul). Estimating gene gain and loss rates in the presence of error in genome assembly and annotation using CAFE 3. *Mol Biol Evol*, *30*(8), 1987-1997.

Hellemans, J., Mortier, G., Paepe, A. D., Speleman, F., & Vandesompele, J. (2007, Jan). qBase relative quantification framework and software for management and automated analysis of real-time quantitative PCR data. *Genome Biol*, *8*(2), R19.

Hughes, J. F., Skaletsky, H., Brown, L. G., Pyntikova, T., Graves, T., Fulton, R. S., ... Page, D. C. (2012, feb). Strict evolutionary conservation followed rapid gene loss on human and rhesus Y chromosomes. *Nature*, *483*(7387), 82-86.

Hughes, J. F., Skaletsky, H., & Page, D. C. (2012, oct). Sequencing of rhesus macaque Y chromosome clarifies origins and evolution of the *DAZ* (Deleted in AZoospermia) genes. *BioEssays*, *34*(12), 1035-1044.

Librado, P., Vieira, F. G., & Rozas, J. (2012, jan). BadiRate: estimating family turnover rates by likelihood-based methods. *Bioinformatics*, *28*(2), 279-281.

Lundrigan, B. L., & Tucker, P. K. (1997, Jul). Evidence for multiple functional copies of the male sex-determining locus, Sry, in African murine rodents. *J Mol Evol*, *45*(1), 60–5.

Maddison, D. R., & Maddison, W. P. (2005). *MacClade 4: Analysis of phylogeny and character evolution.* Sinauer Associates Inc., Sunderland, MA. Retrieved from http://macclade.org (4.08a)

McBrearty, S., & Jablonski, N. G. (2005). First fossil chimpanzee. *Nature*, *437*(7055), 105–108.

Nuytten, H., Wlodarska, I., Nackaerts, K., Vermeire, S., Vermeesch, J., Cassiman, J.-J., & Cuppens, H. (2009, may). Accurate determination of copy number variations (CNVs): Application to the α- and β-defensin CNVs. *J Immunol Methods*, *344*(1), 35-44.

Pagel, M. (1994, jan). Detecting correlated evolution on phylogenies: A general method for the comparative analysis of discrete characters. *Proc R Soc B*, *255*(1342), 37-45.

Perelman, P., Johnson, W. E., Roos, C., Seuánez, H. N., Horvath, J. E., Moreira, M. A. M., ... Pecon-Slattery, J. (2011, Mar). A molecular phylogeny of living primates. *PLoS Genet*, *7*(3), e1001342.

Perne, A., Zhang, X., Lehmann, L., Groth, M., Stuber, F., & Book, M. (2009, dec). Comparison of multiplex ligation-dependent probe amplification and real-time PCR accuracy for gene copy number quantification using the β-defensin locus. *BioTechniques*, *47*(6), 1023-1028.

Rambaut, D., & Drummond, A. J. (2003-2009). *Tracer: MCMC trace analysis tool.* Retrieved from http://beast.bio.ed.ac.uk/Tracer (1.5.0)

Ruijter, J. M., Pfaffl, M. W., Zhao, S., Spiess, A. N., Boggy, G., Blom, J., ... others (2013). Evaluation of qPCR curve analysis methods for reliable biomarker discovery: bias, resolution, precision, and implications. *Methods*, *59*(1), 32–46.

Scally, A., Dutheil, J. Y., Hillier, L. W., Jordan, G. E., Goodhead, I., Herrero, J., ... Durbin, R. (2012, Mar). Insights into hominid evolution from the gorilla genome sequence. *Nature*, *483*(7388), 169–75.

Skaletsky, H., Kuroda-Kawaguchi, T., Minx, P. J., Cordum, H. S., Hillier, L., Brown, L. G., ... Page, D. C. (2003, jun). The male-specific region of the human Y chromosome is a mosaic of discrete sequence classes. *Nature*, *423*(6942), 825-837.

Tosi, A. J., Morales, J. C., & Melnick, D. J. (2000, Nov). Comparison of Y chromosome and mtDNA phylogenies leads to unique inferences of macaque evolutionary history. *Mol Phylogenet Evol*, *17*(2), 133-144.

Tuomi, J. M., Voorbraak, F., Jones, D. L., & Ruijter, J. M. (2010). Bias in the Cq value observed with hydrolysis probe based quantitative PCR can be corrected with the estimated PCR efficiency value. *Methods*, *50*(4), 313–322. Retrieved from http://www.hartfaalcentrum.nl/index.php?main=files&sub=LinRegPCR

Untergasser, A., Cutcutache, I., Koressaar, T., Ye, J., Faircloth, B. C., Remm, M., & Rozen, S. G. (2012, aug). Primer3-new capabilities and interfaces. *Nucleic Acids Res*, *40*(15), e115-e115. Retrieved from http://www.bioinformatics.nl/cgi-bin/primer3plus/primer3plus.cgi/

Wagenmakers, E.-J., & Farrell, S. (2004, Feb). AIC model selection using Akaike weights. *Psychon Bull Rev*, *11*(1), 192-6.

Wallis, M. C., Waters, P. D., & Graves, J. A. M. (2008, Oct). Sex determination in mammals–before and after the evolution of SRY. *Cell Mol Life Sci*, *65*(20), 3182–95.

Ye, J., Coulouris, G., Zaretskaya, I., Cutcutache, I., Rozen, S., & Madden, T. L. (2012). Primer-BLAST: a tool to design target-specific primers for polymerase chain reaction. *BMC Bioinformatics*, *13*(1), 134. Retrieved from http://www.ncbi.nlm.nih.gov/tools/primer-blast/

Zhang, X., ller, S. M., ller, M. M., Huse, K., Taudien, S., Book, M., ... Groth, M. (2014, jan). 8p23 beta-defensin copy number determination by single-locus pseudogene-based paralog ratio tests risk bias due to low-frequency sequence variations. *BMC Genomics*, *15*(1), 1-11.

Zuker, M. (2003, jul). Mfold web server for nucleic acid folding and hybridization prediction. *Nucleic Acids Res*, *31*(13), 3406-3415. Retrieved from http://mfold.rna.albany.edu/?q=mfold

Supplementary Tables

Table S1: Summary of the model fits to the msrY singleton data set alone.

Model	logLik	df	Estimate(s)*	95% CI	BIC	w(BIC)
L	-57.1	1	$\lambda = 0.0252$	0.012–0.048	116	0
L+het	-51.8	2	$\lambda_R = 0.00875, \lambda_{HC} = 0.101$	0.002-0.025, 0.038-0.218	108	0
$L = I$	-49.8	1	$\lambda' = 0.0258$	0.01-0.059	102	0.003
$L = I$+het	-43.2	2	$\lambda'_R = 0.00267, \lambda'_{HC} = 0.104$	0-0.017, 0.038-0.25	90.9	0.777
BD	-51.1	2	$b = 0, d = 0.051$	0-0.03, 0.023-0.098	106	0
BD+het	-45.7	4	$b_R = 0, d_R = 0.0166$<br>$b_{HC} = 0, d_{HC} = 0.193$	0-0.039, 0.003-0.05<br>0-0.167, 0.075-0.394	100	0.007
$DB = I$	-49.7	2	$b' = 0.0178, d = 0.032$	0-0.074, 0.004-0.082	104	0.001
$DB = I$+het	-42.3	4	$b'_R = 0.00279, d_R = 0$<br>$b'_{HC} = 0.0618, d_{HC} = 0.173$	0-0.031, 0-0.026<br>0.007-0.202, 0.046-0.397	93.4	0.211

* The units of estimated parameter values are 'per gene copy per million generations' for b, d, and λ, or 'per gene family per million generations' for i.

Table S2: Summary of the model fits to the msrY AG data set alone.

Model	logLik	df	Estimate(s)*	95% CI	BIC	ΔBIC	w(BIC)
L	-71.4	1	$\lambda = 0.154$	0.092-0.251	145	1.60	0.117
L+het	-70.5	2	$\lambda_R = 0.128, \lambda_{HC} = 0.347$	0.067-0.226, 0.126-1.084	146	2.34	0.081
$L = I$	-70.6	1	$\lambda' = 0.163$	0.094-0.277	144		0.260
$L = I$+het	-69.7	2	$\lambda'_R = 0.134, \lambda'_{HC} = 0.357$	0.066-0.248, 0.102-1.687	144	0.744	0.179
BD	-70.9	2	$b = 0.122, d = 0.185$	0.046-0.233, 0.099-0.3	147	3.14	0.054
BD+het	-68.7	4	$b_R = 0.0161, d_R = 0.161$<br>$b_{HC} = 0.714, d_{HC} = 0.355$	0-0.158, 0.086-0.267<br>0.127-1.476, 0.089-1.074	148	3.83	0.038
$DB = I$	-70.3	2	$b' = 0.14, d = 0.187$	0.054-0.267, 0.089-0.319	146	1.94	0.098
$DB = I$+het	-67.2	4	$b'_R = 0.0184, d_R = 0.176$<br>$b'_{HC} = 0.762, d_{HC} = 0.24$	0-0.147, 0.089-0.306<br>0.238-2.393, 0-1.981	145	0.832	0.172

* The units of estimated parameter values are 'per gene copy per million generations' for b, d, and λ, or 'per gene family per million generations' for i.

Table S3: List of rhesus macaque BACs used for primer design.

GenBank accessions
AC206800, AC207040,<br>AC207520, AC208129,<br>AC208130, AC208132,<br>AC208133, AC208822,<br>AC209262, AC209263,<br>AC209264, AC212487,<br>AC212790, AC214069,<br>AC215549, AC215550,<br>AC215640, AC216894,<br>AC217105, AC217129,<br>AC217130, AC217138,<br>AC219066, AC225627,<br>AC225636, AC225837,<br>AC231654, AC231831,<br>AC232761, AC234329,<br>AC234330, AC237223

Table S4: Quality of the genomic DNA extracts for each sample.

Sample	Species	Set(s)*	Eluted in	Nucleic Acid Conc. (ng/μL)	Dilution	Purity (A_{260}/A_{280})
Zoo	*M. mulatta*	1st	dH_2O	20.0	1	1.74
Zoo	*M. mulatta*	2nd	dH_2O	20.0	0.125	1.74
143	*M. arctoides*	2nd	dH_2O	251.2	0.067	1.75
P001	*M. maura*	2nd	dH_2O	184.3	0.067	1.84
PM616	*M. maura*	2nd	dH_2O	16.6	0.5	1.73
PM545	*M. tonkeana* east	1st	dH_2O	8.8	1	1.87
PM545	*M. tonkeana* east	2nd	Buf. AE	13.4	0.067	1.97
PM561	*M. tonkeana* west	1st	dH_2O	10.0	1	1.73
PM561	*M. tonkeana* west	2nd	Buf. AE	17.4	0.067	1.90
PM582	*M. tonkeana* west	1st	dH_2O	1.6	1	1.32
PM582	*M. tonkeana* west	2nd	Buf. AE	7.6	0.5	1.60
PM604	*M. tonkeana* west	1st	dH_2O	4.8	1	1.01
PM604	*M. tonkeana* west	2nd	dH_2O	18.8	0.25	1.95
PM638	*M. hecki*	1st	dH_2O	5.1	1	1.83
PM638	*M. hecki*	2nd	Buf. AE	7.8	0.5	1.78
PM1014	*M. hecki*	$1^{st} + 2^{nd}$	dH_2O	4.6	1	2.03
PM655	*M. nigrescens*	1st	dH_2O	8.3	1	1.38
PM655	*M. nigrescens*	2nd	dH_2O	13.8	0.5	1.81
PM661	*M. nigra*	$1^{st} + 2^{nd}$	dH_2O	1.4	1	1.76
PM665	*M. nemestrina*	1st	dH_2O	7.8	1	1.80
PM665	*M. nemestrina*	2nd	dH_2O	9.4	0.5	1.91
PM704	*M. ochreata*	1st	dH_2O	2.9	1	1.73
PM704	*M. ochreata*	2nd	dH_2O	11.5	0.25	1.97

* 1st refers to the set of preliminary assays performed using the iTaq Fast SYBR Green Supermix with ROX; 2nd refers to the set of assays performed using the SsoFast EvaGreen Supermix (see the "qPCR" subsection of the Supplementary Methods section); $1^{st} + 2^{nd}$ refers to both sets of assays.

Table S5: Accession numbers for sequences used to build gene trees but not sequenced in this study.

Gene	Species	Genbank accession number
AMELY	*H. sapiens*	NC_000024.10: 6 868 563-6 867 999
AMELY	macaques	HM071684-HM071693
AMELY	*P. troglodytes*	AB091782.1
CDY	*C. jacchus*	FJ526998.1
DAZ1	*H. sapiens*	NC_000003.12
DAZ1	*M. mulatta*	AF053608.1
DAZ1	*P. troglodytes*	AF053606.1
DBY	*C. jacchus*	AC245293.3: 42 530-42 764, 60 146-60 340
DBY	*H. sapiens*	NC_000024.10: 12916651-12916854
DBY	macaques	HM071810-HM071817
DBY	*P. troglodytes*	AC146254.2: 47 245-47 042
PRKY	*H. sapiens*	NC_000024.10: 7 325 834-7 326 451
PRKY	macaques	HM071789-HM071798
PRKY	*P. troglodytes*	NC_006492.3: 25 186 998-25 186 381
RBMY	*C. jacchus*	AC220987.3
SMCY	*C. jacchus*	AC226175.3: 2 297-3 036
SMCY	*H. sapiens*	NC_000024.10: 19 707 809-19 707 071
SMCY	macaques	HM071647-HM071656
SMCY	*P. troglodytes*	AC144429.1: 66 765-66 039
SRY	*C. jacchus*	AC221052.5: 165 915-166 709
SRY	*H. sapiens*	NM_003140.2
SRY	macaques	HM071767-HM071776
SRY	*P. troglodytes*	JF293177.1
TBL1Y	*H. sapiens*	NC_000024.10: 7 070 164-7 070 931
TBL1Y	macaques	HM071704-HM071713
TBL1Y	*P. troglodytes*	AC146484.3: 22 321-21 554
TSPY	*C. jacchus*	AC234250.2
TSPY	*M. nigrescens*	AF284268.2
TSPY	*M. nigra*	AF284267.2
TSPY	*M. hecki*	AF284256.2

Continued on next page...

Table S5 – Continued from previous page

**Gene**	**Species**	**Genbank accession number**
TSPY	*M. tonkeana* east	AF284236.2
TSPY	*M. tonkeana* west	AF284235.2
TSPY	*M. ochreata*	AF284269.2
TSPY	*M. maura*	AF284257.2
USP9Y	*C. jacchus*	AC234102.2: 7 5193-7 5979
USP9Y	*H. sapiens*	NM_004654.3
USP9Y	macaques	HM071667-HM071676
USP9Y	*P. troglodytes*	NM_001009110.1
UTY	*C. jacchus*	AC231992.2: 2 967-3 707
UTY	*H. sapiens*	XM_011531442.1
UTY	macaques	HM071726-HM071733
UTY	*P. troglodytes*	AC146194.2: 47 615-46 872
XKR3	*H. sapiens*	NM_175878.3
ZFY	*C. jacchus*	AC221058.4: 151 603-152 290
ZFY	*H. sapiens*	NM_003411.3
ZFY	macaques	HM071746-HM071755
ZFY	*P. troglodytes*	NM_001009003.1

Table S6: qPCR target information

**Primer Name**	**Length** (nt)	**Primer Location*** (bp)	**Exon/ Intron**	**Specificity Check**
qCDY_For1<br>qCDY_Rev1	132	8813798-79, 8903851-70<br>8813693-66, 8903956-83	ex1	direct sequencing
qDAZ_A_For1<br>qDAZ_A_Rev1	103	10637794-814, 10650686-706<br>10637877-97, 10650769-89	ex4-ex6	restriction enzyme: CviAII
qDAZ_BCD_For2<br>qDAZ_BCD_Rev2	117	9166093-74, 9177331-12, 9186075-56<br>9165995-76, 9177233-14, 9185977-58	ex4-ex6	direct sequencing
qHSFY_A_For1<br>qHSFY_A_Rev1	135	8386429-48, 8553596-77<br>8386545-64, 8553480-61	ex2	restriction enzyme: CviAII
qHSFY_B_For1<br>qHSFY_B_Rev1	146	8006912-893<br>8006785-66	ex2	restriction enzyme: MboI
qRBMYex3_For1<br>qRBMYex3_Rev3	93	6278470-50<br>6278377-98	ex3	direct sequencing
qSRY_For1<br>qSRY_Rev1	91	81 721-02<br>81 649-30	ex1	none
qTSPY_B_For3<br>qTSPY_B_Rev3	124	6308032-13<br>6307929-08	ex3-ex5	restriction enzyme: Fnu4HI
qXKRY_For1<br>qXKRY_Rev1	120	7642396-77<br>7642295-76	ex1	restriction enzyme: DdeI

* On the rhesus macaque Y-chromosome.

Table S7: Table of qPCR conditions

**Assay**	**Set***	**Annealing Temp.** (°C)	**BSA Added?**	**For. Primer Vol.** (μL)	**Rev. Primer Vol.** (μL)
CDY	2nd	59.0	yes	0.600	1.000
DAZa	1st	61.4	no	0.323	0.323
DAZa	2nd	61.4	no	0.833	0.833
DAZbcd	2nd	59.0	yes	1.000	0.600
HSFYa	1st	63.4	no	0.323	0.323
HSFYa	2nd	63.4	yes	0.833	0.833
HSFYb	1st	63.4	no	0.323	0.323
HSFYb	2nd	63.4	yes	0.500	0.500
RBMY	2nd	56.0	yes	0.300	0.300
SRY	1st	63.8	no	0.323	0.323
SRY	2nd	63.4	yes	0.833	0.833
TSPYb	1st	63.4	no	0.323	0.323
TSPYb	2nd	63.4	yes	0.300	0.300
XKRY	1st	63.4	no	0.323	0.323
XKRY	2nd	63.4	yes	0.300	0.500

* 1st Refers to the set of preliminary assays performed using the iTaq Fast SYBR Green Supermix with ROX; 2nd refers to the set of assays performed using the SsoFast EvaGreen Supermix (see the "qPCR" subsection of the Supplementary Methods section).

Table S8: Table of qPCR validation.

Assay	Set*	Estimated % Eff.†	LOD‡	C_q St. Dev. at LOD	Mean Eff.[1]	Among Sample Eff. Variation[2]
CDY	2nd	109.3	$2.06 \cdot 10^{-3}$	0.554	1.756	0.022
DAZa	1st	93.2	$1.95 \cdot 10^{-3}$	0.213	1.822	0.061
DAZa	2nd	107.7	$1.13 \cdot 10^{-3}$	0.0917	1.683	0.034
DAZbcd	2nd	101.0	$2.06 \cdot 10^{-3}$	0.198	1.634	0.027
HSFYa	1st	90.2	$1.95 \cdot 10^{-3}$	0.119	1.714	0.044
HSFYa	2nd	102.7	$2.06 \cdot 10^{-3}$	0.199	1.791	0.035
HSFYb	1st	103.0	$1.95 \cdot 10^{-3}$	0.670	1.781	0.054
HSFYb	2nd	109.9	$5.50 \cdot 10^{-3}$	0.193	1.778	0.018
RBMY	2nd	106.8	$1.03 \cdot 10^{-3}$	1.45	1.701	0.048
SRY	1st	90.5	$6.91 \cdot 10^{-4}$	NA	1.825	0.070
SRY	2nd	99.4	$2.06 \cdot 10^{-3}$	0.161	1.815	0.030
TSPYb	1st	91.1	$6.91 \cdot 10^{-4}$	1.27	1.761	0.025
TSPYb	2nd	102.9	$1.03 \cdot 10^{-3}$	0.320	1.658	0.037
XKRY	1st	97.7	$6.91 \cdot 10^{-4}$	1.51	1.873	0.020
XKRY	2nd	106.3	$3.92 \cdot 10^{-2}$	0.391	1.666	0.035

* 1st Refers to the set of preliminary assays performed using the iTaq Fast SYBR Green Supermix with ROX; 2nd refers to the set of assays performed using the SsoFast EvaGreen Supermix (see the "qPCR" subsection of the Supplementary Methods section).

† Reaction efficiency as estimated from an eight-point dilution gradient using CFX Manager Software.

‡ LOD is expressed as the dilution of the *M. mulatta* gDNA sample listed in Table S4.

[1] Mean reaction efficiency as estimated from averaging the efficiencies determined for each qPCR reaction performed on experimental and reference samples using LinRegPCR.

[2] Standard deviation of the mean LinRegPCR reaction efficiencies of each sample for each assay.

Table S9: Sequences of sequencing and qPCR primers and their annealing temperatures for PCR.

Primer Name*	Gene(s)†	Sequence	Annealing Temp. (°C)
CDY_For340	*CDY* 1-2	GCCAGCAAGAACGTTAGGAG	58
CDY_Rev892	*CDY* 1-2	TCTGGGTGAATCCATCCTCT	58
CDY_For1005	*CDY* 1-2	CAGTGCAGCTGGAAGTGTGT	58
CDY_Rev1308	*CDY* 1-2	CCTTTTCTGTTGCCCACACT	58
CDY_Rev1536	*CDY* 1-2	TCTCTCATTGGCCTTTTCCA	58
CDYps_For1	*CDY* ψ	CCAGTCAGGGATGCTTTCTC	58
CDYps_Rev1	*CDY* ψ	GGCCCTTTCCAACTCAATCT	58
qCDY_For1	*CDY*	GCGGTCTTGATTTTGGGTAT	58
qCDY_Rev1	*CDY*	ACTGATACAACAATAGGCTTTTTAAACT	58
DAZex4-6_For1	*DAZ2*	CCTTTATAGCTATGGATTTGTTTCA	57
DAZex4-6_Rev1	*DAZ2*	GCAGTTCTCACCTGAACGTACT	57
DAZex4-6_For2	*DAZ1*	CTGGACTATGTGCTGTATGATGG	58
DAZex4-6_Rev2	*DAZ1*	TTACAGGATTCAGCGTTATTGG	58
DAZex4-6_For3	*DAZ1*	CTTCACCTTTTCTCTGCCTTT	57
DAZex4-6_Rev3	*DAZ1*	ACAGGATTCAGCGTTATTGG	57
qDAZ_A_Rev2	*DAZ*	TCCAGACATTCTGAAACTGC	58
qDAZ_A_For1	*DAZ2*	TCAGTCACAGATCCATATCCA	59
qDAZ_A_Rev1	*DAZ2*	CACGTGTCAAAAAGAACAATG	59
qDAZ_BCD_For2	*DAZ1*	CTGCAATCAGGAAACAAAAA	58
qDAZ_BCD_Rev2	*DAZ1*	CGAGCACCTTATAAAAAGCA	58
HSFYa_For1	*HSFY2-3*	CTGGAACAGCGGCTAAAGA	57
HSFYa_Rev1	*HSFY2-3*	CTTGTTGGAACAGCAGGTGA	57
HSFYb_For1	*HSFY1*	GCCTGGAAGAGTAGCTCAGG	57
HSFYb_For2	*HSFY1*	CATGCAGCCTGGAAGAGTAG	57
HSFYb_Rev1	*HSFY1*	GGGCCAGATGAATTAGCAGT	57
HSFYintron_For	*HSFY1-3*	GGGATGAGAATGGAACTTGC	57
HSFYintron_Rev	*HSFY1-3*	CCATGTTAGCCCCTGCTCTA	57
HSFYex1_For	*HSFY1-3*	AAGCYTCCAMTAGGTCTCCA	55
HSFYex1_Rev	*HSFY1-3*	GAAAGGTGGSTASAAAGGCAGA	55
HSFYex1_For2	*HSFY2-3*	AGGTCTCCATTGTGTGAGCA	58
HSFYex1_Rev2	*HSFY2-3*	CAGCCAGAAAGGTGGGTAGA	58
HSFYex1_For3	*HSFY1*	GCTTCCAATAGGTCTCCATTGT	58

Continued on next page...

Table S9 – Continued from previous page

Primer Name*	Gene(s)†	Sequence	Annealing Temp. (°C)
HSFYex1_Rev3	*HSFY1*	CTGCCAGAAAGGTGGCTACA	58
HSFYex1ps1For	*HSFY* ψ	TCCCCTAGGTCTCCATTGTG	55
HSFYex1ps1Rev	*HSFY* ψ	GTTGGCCAAAGAAGCAGAAG	55
HSFYex1ps2For	*HSFY* ψ	TCTCCATTGCGTGAACACAT	55
HSFYex1ps2Rev	*HSFY* ψ	GCCAGAAAGTTGACTAGAAAAGC	55
HSFYex2For	*HSFY1-3*	TGGCTGTCCCCAACTTTTAG	58
HSFYex2Rev	*HSFY1-3*	GTTGARKAGCTGGCCTGGAA	58
HSFYex2_Rev2	*HSFY2-3*	TCCAGTGGTGATGGTTGAGT	58
HSFYex2_Rev3	*HSFY1*	TGTCCAGTAGTGATGGTTGAAGA	58
HSFYex2psFor	*HSFY* ψ	TTCAAATGTGGCTGTCTCCA	58
HSFYex2psRev	*HSFY* ψ	TGGTTGAAGAGTTGGCCTGT	58
qHSFYex2_A_For1	*HSFY2-3*	AGAATCACCTGCTGTTCCAA	58
qHSFYex2_A_Rev1	*HSFY2-3*	CCTGGAAAGAGGGCTAGATG	58
qHSFYex2_B_For1	*HSFY1*	TGGCCCAATTAGAAGTGGTT	58
qHSFYex2_B_Rev1	*HSFY1*	CCCCTTGCTTGCATATAGGT	58
PRYex3F	*PRY* ψ	TGGGAAGGTTGGCTCTATTT	55
PRYex3R	*PRY* ψ	CACGAGGTACCCTGAAAACA	55
PRYex3_For2	*PRY* ψ	TTCAAGGTATGGGAAGGTTGA	57
PRYex3_Rev2	*PRY* ψ	GGTGTCCCAAAGCCACAG	57
PRYex3_For3	*PRY* ψ	AGGTGTCCCAAAGCTGTGAT	57
PRYex3_Rev3	*PRY* ψ	TCCAGTGGTGATGGTTGAGT	57
RBMYex1-2_For2	*RBMY*	GCAGCACAATGGTAGAAGCA	58
RBMYex1-2_Rev3	*RBMY*	GGCATTTTACTATCTTCAAAAGTTACA	58
RBMYex1-2_For1	*RBMY* ψ	AGCTTTTCATTGGTGGGCTA	58
RBMYex1-2_Rev1	*RBMY* ψ	ATCTGCAGGGTTCTCAAACG	58
RBMYex1-2_For4	*RBMY* ψ	TGGCAAGCTTTTCATTAGCA	58
RBMYex1-2_Rev4	*RBMY* ψ	GCTTTGGCAGCATTCTTAGC	58
RBMYex3_For3	*RBMY*	GAACAAGCCAAGAAACCATCA	58
RBMYex3_Rev3	*RBMY*	AAACATTACCCAAGTGTCCTTCA	58
RBMYex3_For1	*RBMY* ψ	AACAAGCCAACAAACCATCC	58
RBMYex3_Rev1	*RBMY* ψ	CATTACCCAAGTGTCCTCCA	58
RBMYex3_For2	*RBMY* ψ	AGTCTTTGGATGGAAAAGCAA	58
RBMYex3_Rev2	*RBMY* ψ	GTCCTTCATGTGAGGGATGC	58
RBMYex3_For4	*RBMY* ψ	ATGCCAATGTTATTGATAGTATGC	58

Continued on next page...

Table S9 – Continued from previous page

**Primer Name***	**Gene(s)†**	**Sequence**	**Annealing Temp.** (°C)
RBMYex3_Rev4	*RBMY* ψ	TTCATGTGAGGGAAGCCATC	58
RBMYex3_For5	*RBMY* ψ	TGCTTAGGTTAAATATGCCAATG	58
RBMYex3_Rev5	*RBMY* ψ	GTGAGGGAAGCCATCCTCTT	58
RBMYex3_For6	*RBMY* ψ	CCACAGGTGACATAAATCTGCT	58
RBMYex3_Rev6	*RBMY* ψ	ATCATGTGAGGGAAGCCACT	58
qRBMYex3_For1	*RBMY*	CATTTCAAAGTGGTGGTAGGC	58
qRBMYex3_Rev3	*RBMY*	CCTCCACTACTTCCTTTTGCAG	58
qSRY_For1	*SRY*	CTCAAGAATGCAGCACCAGT	58
qSRY_Rev1	*SRY*	GCTTTGTCGAGTGGCTGTAG	58
TSPYex3-5For	*TSPY1-5*	GTGAAAGAAGCGAAGCATCC	58
TSPYex3-5Rev	*TSPY1-5*	CTCTTCAGGYGGCTTCATC	58
TSPYex3-5_Rev_a1	*TSPY2-5*	TATCCCGGGTATCAGACAGC	58
TSPYex3-5_Rev_b1	*TSPY1*	CCTCATGTAGCATTGCATGG	58
TSPYex3-5_For_b2	*TSPY1*	CACCTCAGCCAAAAAGGTGT	58
TSPYex3-5_Rev_b2	*TSPY1*	TCAGGGGAATCAATCGAGAG	58
qTSPY_B_For3	*TSPY1*	TCCAATTCAGTGGTGTCAGG	58
qTSPY_B_Rev3	*TSPY1*	CCACCTCAGCAATCCTATTACC	58
XKRY_For28	*XKRY*	CCTGATGACATGTTCCCTGTT	55
XKRY_Rev354	*XKRY*	AGCATCAGTACTGTACCCACCA	55
XKRY_For24	*XKRY* ψ	CATTGCTGATGACATGTTCTCTC	55
XKRY_Rev339	*XKRY* ψ	CCCAACATGCTGGAATTATTTT	55
qXKRY_For1	*XKRY*	ATATGGCGTTTTCTGGAGGT	58
qXKRY_Rev1	*XKRY*	ATGGTGCCAACAATGGTACA	58

* Primers whose names begin with "q" were used for qPCR; all other primers were used only for PCR amplification and sequencing.
† ψ indicates that a msrY-linked, pseudogenized version of the gene is targeted by this primer.

Table S10: Table of C_q values for no template control (NTC) reactions for all assays performed.

**Assay**	**Set***	C_q **Values**†
CDY	2nd	35.45, 36.55, 37.18, 37.93, NA, NA, NA
DAZa	1st	NA, NA, NA
DAZa	2nd	37.21, 37.79, 38.11, Na
DAZbcd	2nd	NA, NA, NA, NA, NA, NA, NA, NA, NA
HSFYa	1st	2.17, NA, NA, NA, NA
HSFYa	2nd	35.82, 36.50, 36.53, 39.59, NA, NA
HSFYb	1st	NA, NA, NA, NA, NA
HSFYb	2nd	NA, NA, NA, NA
RBMY	2nd	36.37, 36.42, 36.43 36.47, 37.82
SRY	1st	NA, NA, NA, NA, NA, NA, NA
SRY	2nd	NA, NA, NA
TSPYb	1st	NA, NA, NA
TSPYb	2nd	NA, NA, NA, NA, NA, NA, NA
XKRY	1st	NA, NA, NA
XKRY	2nd	NA, NA, NA, NA, NA, NA, NA

* 1st Refers to the set of preliminary assays performed using the iTaq Fast SYBR Green Supermix with ROX; 2nd refers to the set of assays performed using the SsoFast EvaGreen Supermix (see the "qPCR" subsection of the Supplementary Methods section).
† "NA" values indicate a C_q that was undetectable.

Table S11: Table of target stability values for reference genes used in qPCR.

**Assay**	**CV**	**M-value**
SRY	0.175	0.358
TSPYb	0.123	0.303
XKRY	0.100	0.285
**Mean**	0.133	0.315

Table S12: Table of intraassay variability, shown as standard errors of normalized relative quantities, for each sample and the mean across all samples.

**Sample**	**CDY**	**DAZa**	**DAZbcd**	**HSFYa**	**HSFYb**	**RBMY**	**XKRY**
PM1014	0.074	0.065	0.077	0.035	0.099	–	0.049
PM638	0.047	0.058	0.055	0.020	0.015	0.051	0.092
PM704	0.036	–	–	0.033	0.031	0.027	0.039
PM655	0.021	0.063	0.039	0.059	0.068	0.041	0.049
PM604	0.063	0.019	0.055	0.031	0.028	–	0.061
PM561	0.066	0.038	0.053	0.034	0.029	–	0.073
PM582	0.046	0.053	0.041	0.039	0.067	–	0.044
PM545	0.074	0.035	0.048	0.055	0.068	0.089	0.052
PM616	0.030	0.040	0.031	0.038	0.056	0.032	0.049
P001	0.080	0.033	0.044	0.068	0.057	0.036	0.051
PM661	0.069	0.037	0.032	0.123	0.058	–	0.058
PM665	0.056	0.049	0.034	0.058	0.047	–	0.054
143	0.274	0.024	0.068	0.067	0.053	0.066	0.085
Zoo	0.218	0.036	0.042	0.028	0.030	0.028	0.035
**Mean**	0.082	0.042	0.048	0.049	0.050	0.046	0.057

Supplementary Figures

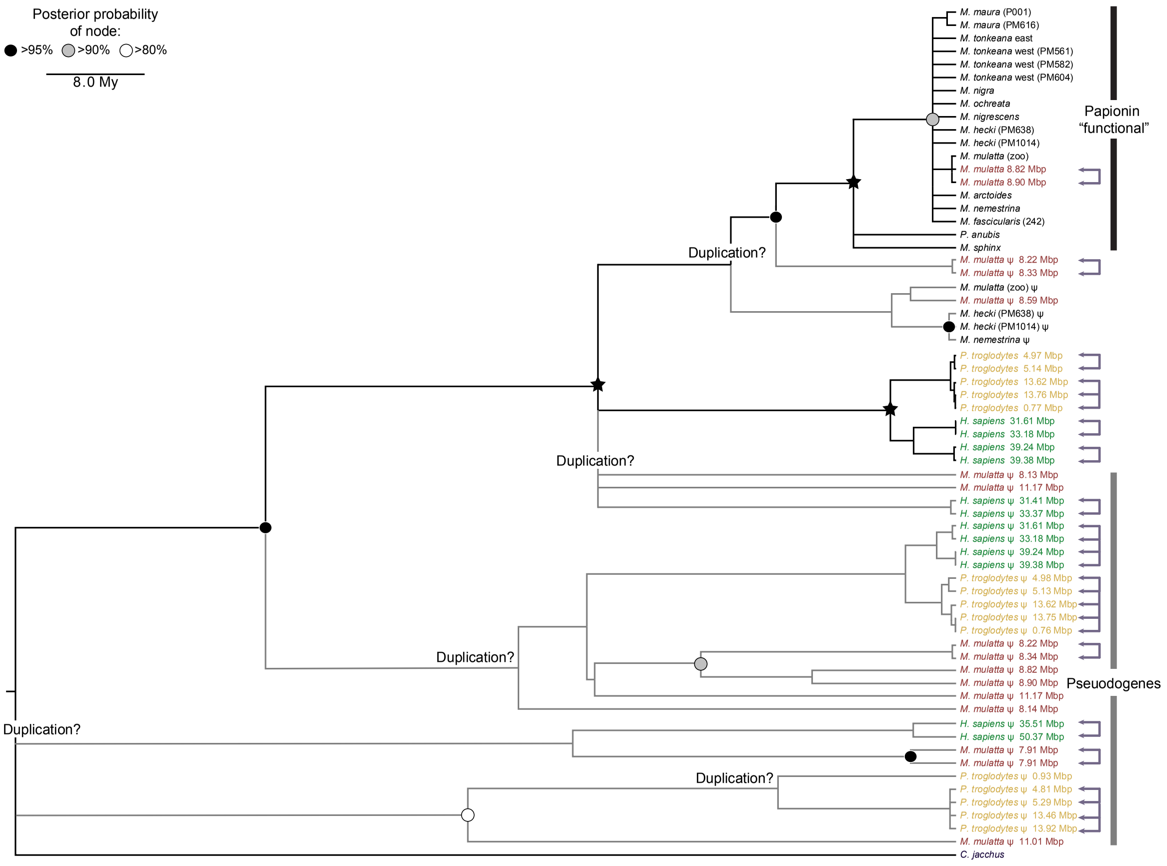

Figure S1: **Maximum clade credibility tree of *CDY*.** Known functional genes (in rhesus, human, and chimp) and presumed functional genes (in marmoset and papionin species) are connected by thick branches. The approximate position, in megabases, on the completed Y-chromosome is given for each rhesus macaque, human, and chimp sequence. All unlabeled nodes have $> 99\%$ posterior probability. Sample ID's can be found in Table S4

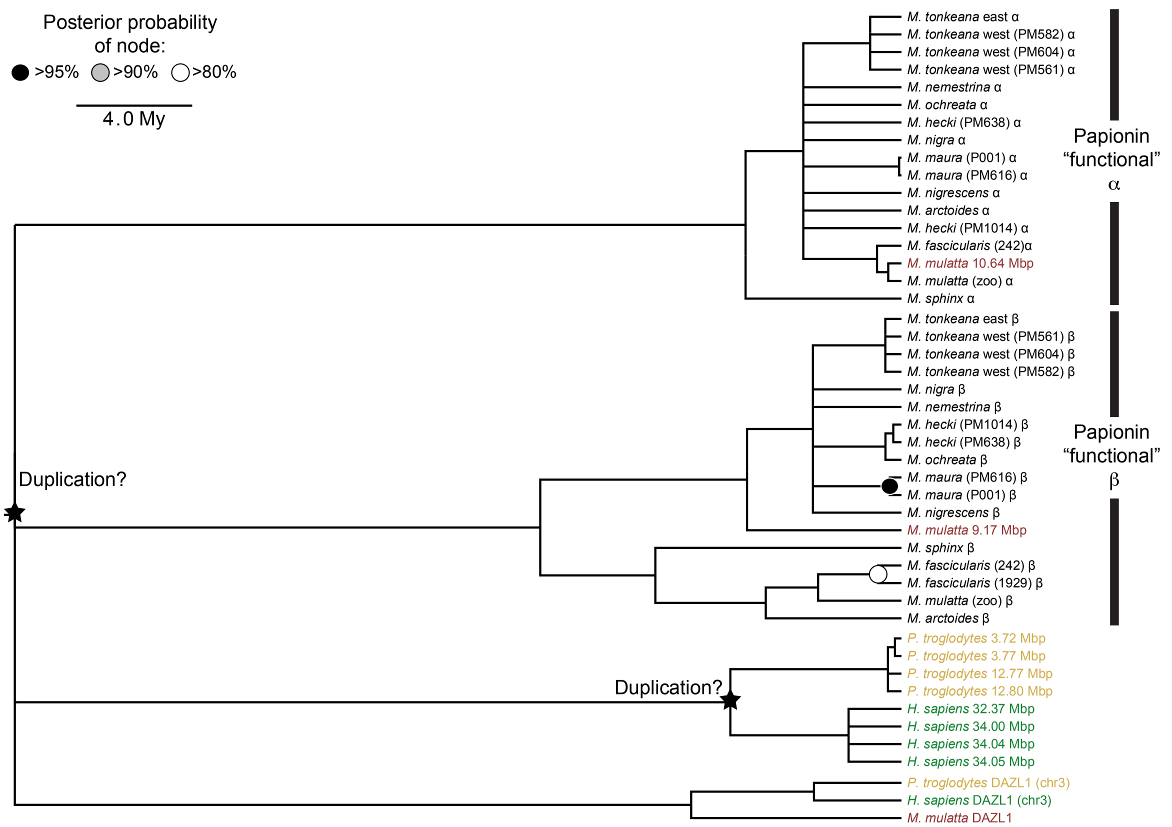

Figure S2: **Maximum clade credibility tree of *DAZ*.** Three orthologs of the autosomal paralog *DAZL1*, which was transposed to the msrY in the Old World primate ancestor (Hughes, Skaletsky, & Page, 2012), are included as an outgroup. Papionin orthologs of rhesus *DAZ1* are labeled “α” and orthologs of *DAZ2* are labeled “β,” corresponding to the primer set (“a” or “bcd,” respectively, in Table S9) that successfully amplified the sequence. Other features of the tree are drawn as detailed in Figure S1.

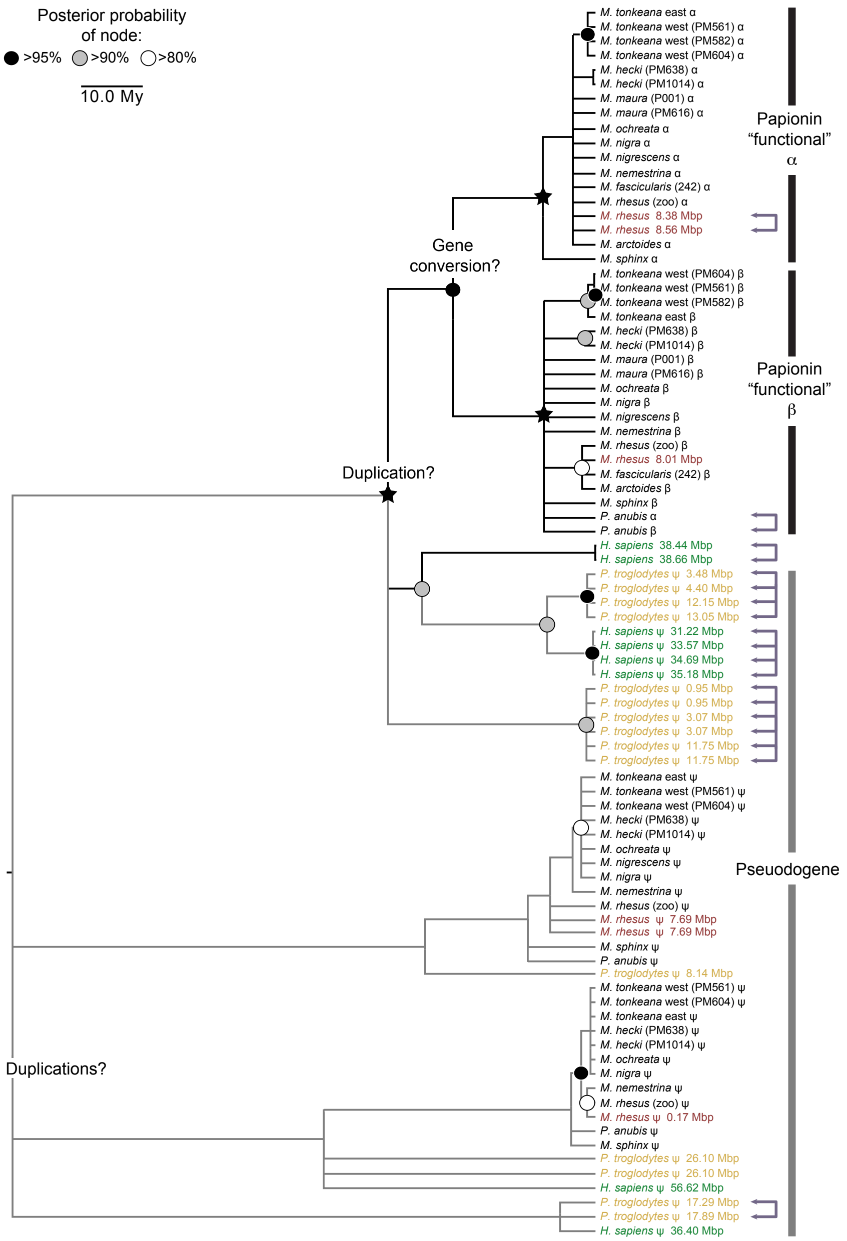

Figure S3: **Maximum clade credibility tree of *HSFY*.** Papionin orthologs of rhesus *HSFY2-3* are labeled "α" and orthologs of *HSFY1* are labeled "β," corresponding to the primer set ("a" or "b," respectively, in Table S9) that was used for sequencing. Other features of the tree are drawn as detailed in Figure S1.

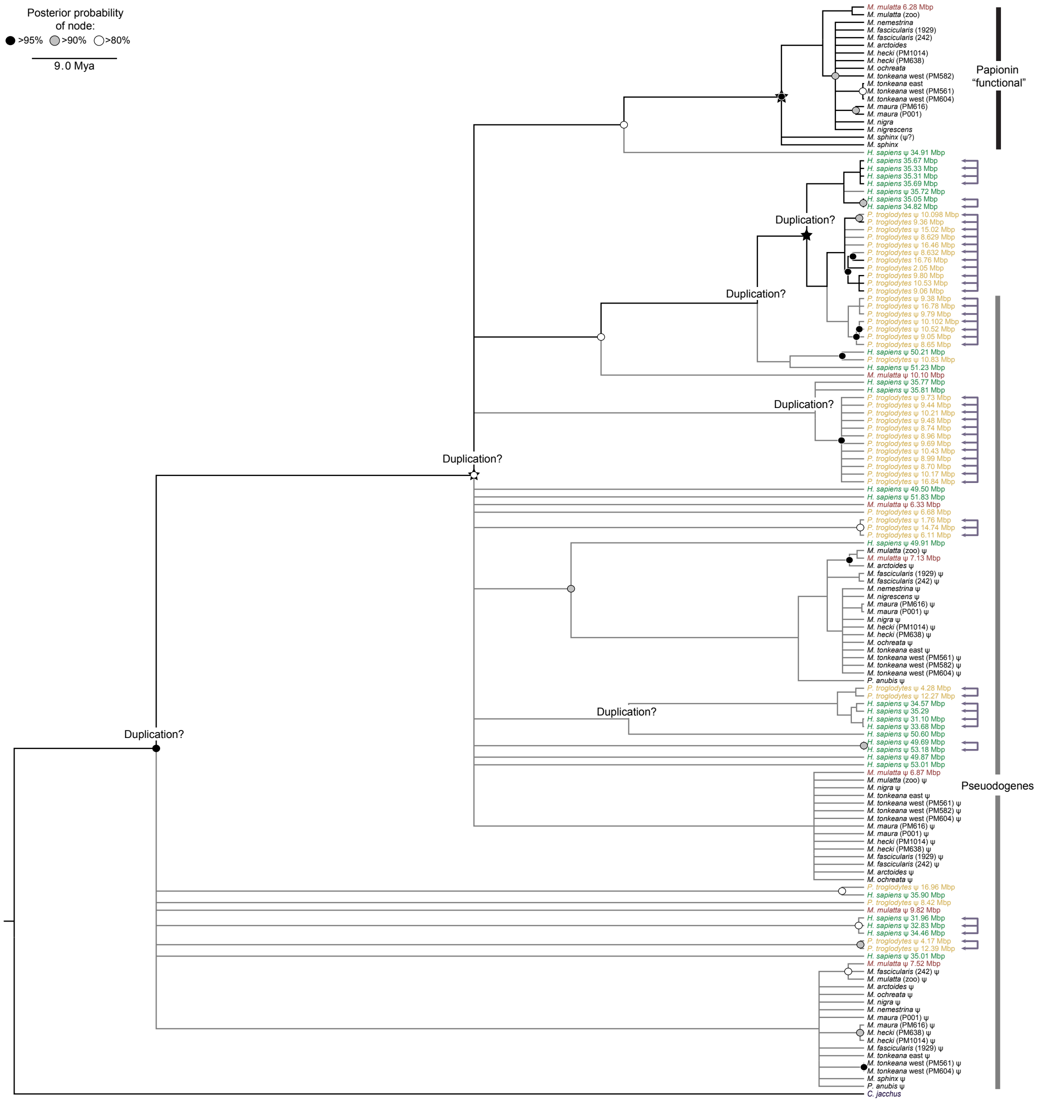

Figure S4: **Maximum clade credibility tree of *RBMY*.** Features of the tree are drawn as detailed in Figure S1.

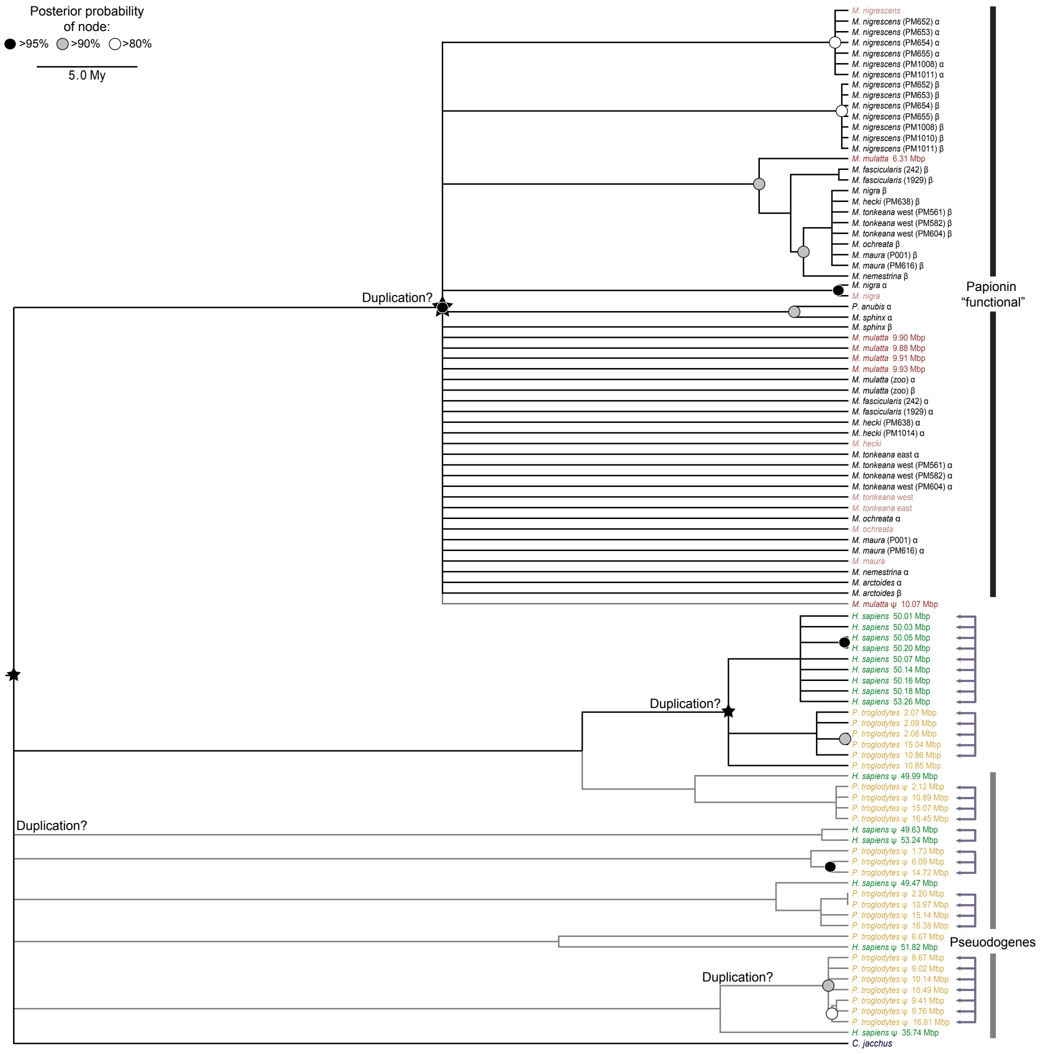

Figure S5: **Maximum clade credibility tree of *TSPY*.** Sulawesi macaque species written in pink were sequenced by Tosi et al. (2000) (see Table S5). Papionin orthologs of rhesus *TSPY2-5* are labeled "α" and orthologs of *TSPY1* are labeled "β," corresponding to the primer set ("a" or "b," respectively, in Table S9) that was used for sequencing. Other features of the tree are drawn as detailed in Figure S1.

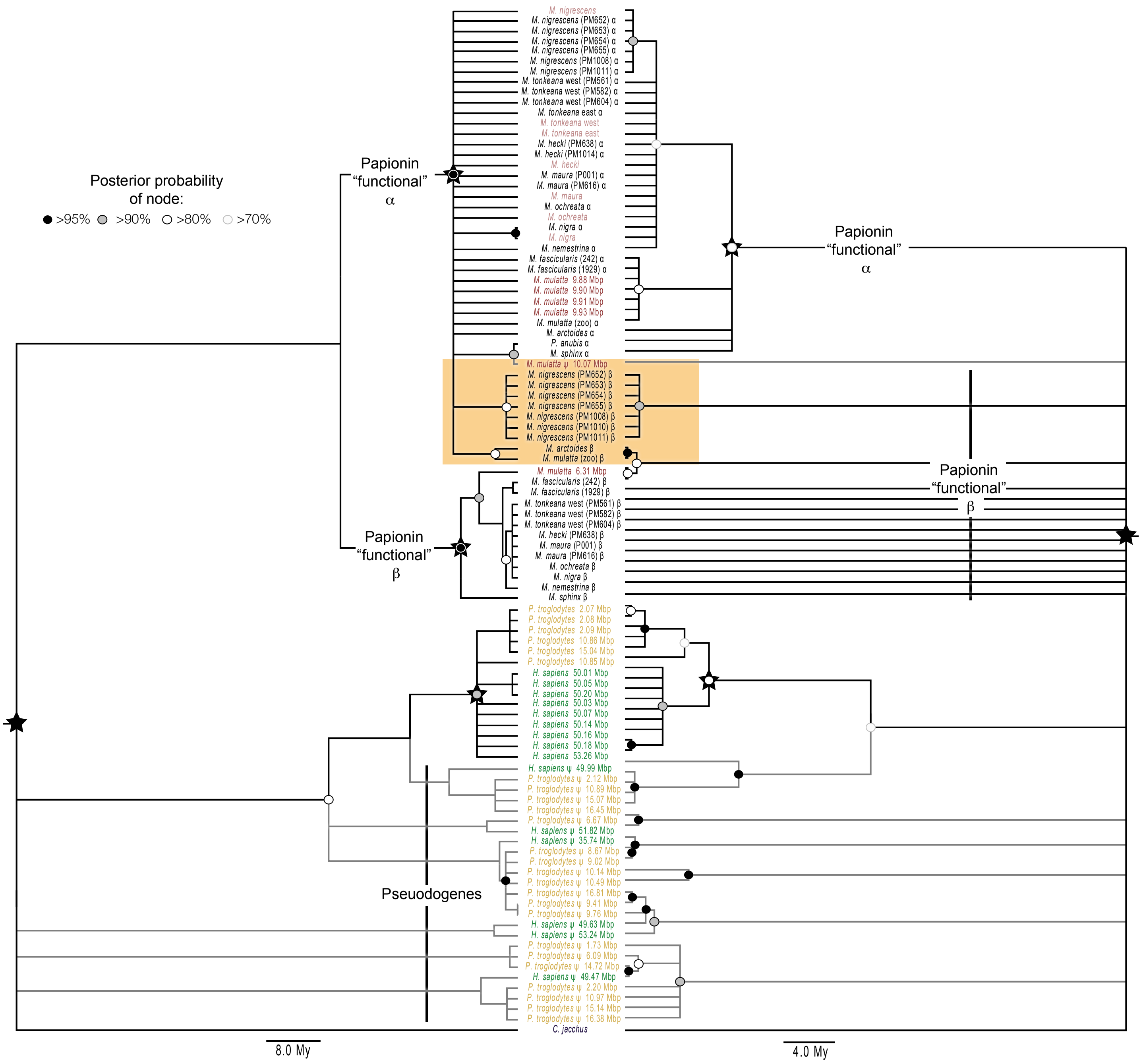

Figure S6: **Maximum clade credibility trees of the 5' half (left) and 3' half (right) of *TSPY*.** The trees are labeled as in Figure S5 except that nodes marked with gray outlined open circles have a posterior probability of $> 70\%$. Partial gene conversion events where the relationships between leaves differ between the 5' and 3' end are highlighted by the yellow box.

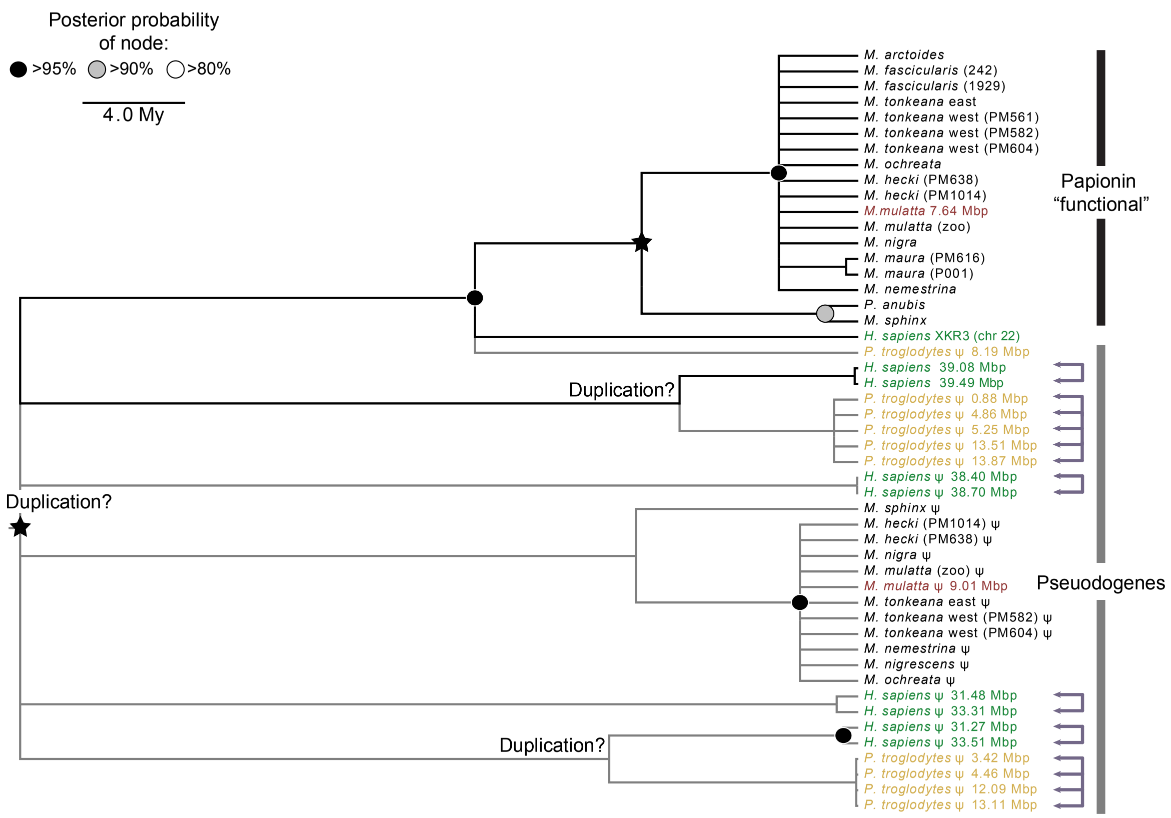

Figure S7: **Maximum clade credibility tree of *XKRY*.** The human autosomal paralog *XKR3*, which was transposed from the msrY to chromosome three in the Old World primate ancestor (Bhowmick et al., 2007), is also included. Other features of the tree are drawn as detailed in Figure S1.

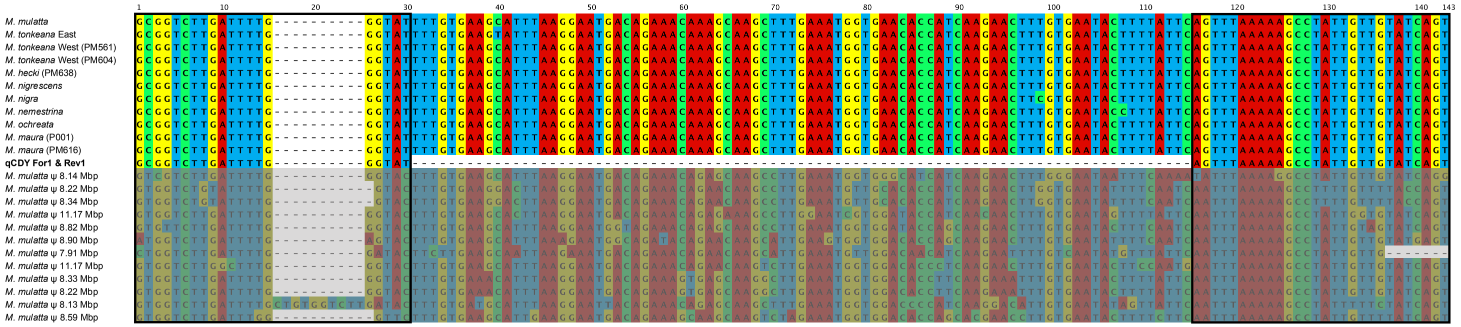

Figure S8: **Alignment of the qPCR amplicon sequence of *CDY* and its primers for select macaque samples.** The sites corresponding to the forward and reverse primer sequences are enclosed in black boxes on the left and right, respectively. Greyed-out sequences below the primers show *CDY* pseudogene sequences from *M. mulatta*, which the primers are designed to *not* amplify.

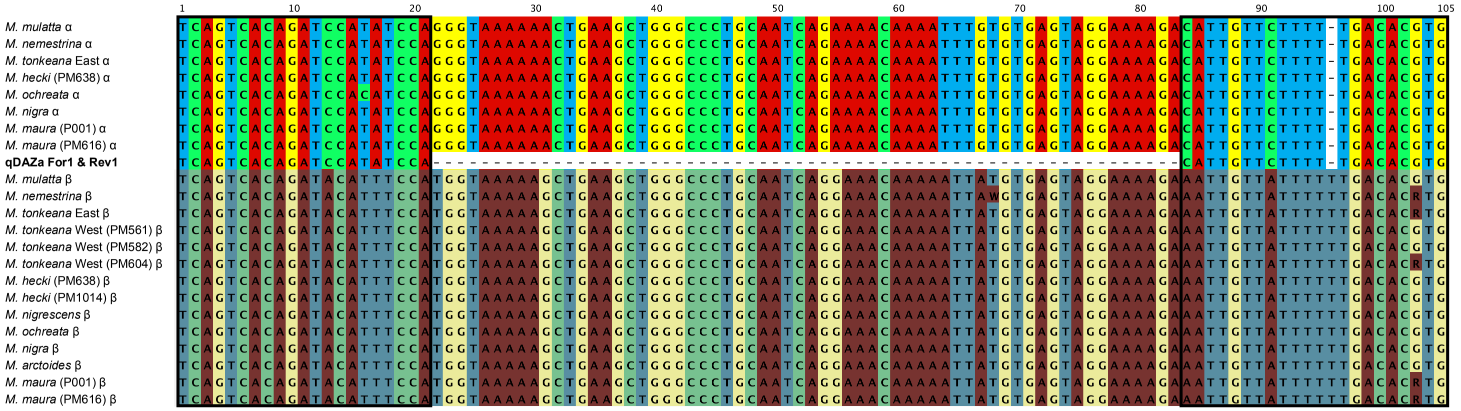

Figure S9: **Alignment of the qPCR amplicon sequence of *DAZ*-a and its primers for select macaque samples.** The sites corresponding to the forward and reverse primer sequences are enclosed in black boxes on the left and right, respectively. Washed-out sequences below the primers show *DAZ*-bcd sequences from selected macaques, which the primers are designed to *not* amplify. The *M. ochreata* sample has a substitution (T→C) at a site six nucleotides from the 5' end of the forward primer that prevented efficient amplification of this assay in this species.

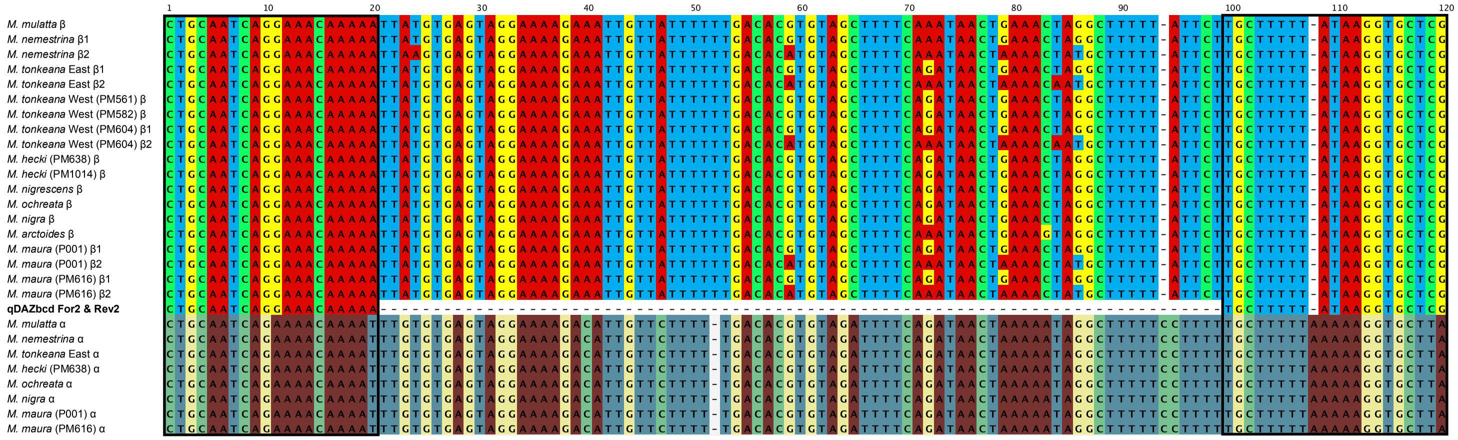

Figure S10: **Alignment of the qPCR amplicon sequence of *DAZ*-bcd and its primers for select macaque samples.** The sites corresponding to the forward and reverse primer sequences are enclosed in black boxes on the left and right, respectively. Washed-out sequences below the primers show *DAZ*-a sequences from selected macaques, which the primers are designed to *not* amplify. Some samples (e.g. *M. nemestrina* and *M. tonkeana* East) exhibited sequence differences between exon repeats; different sequenced haplotypes are shown for these samples and labeled "$\beta 1$" and "$\beta 2$."

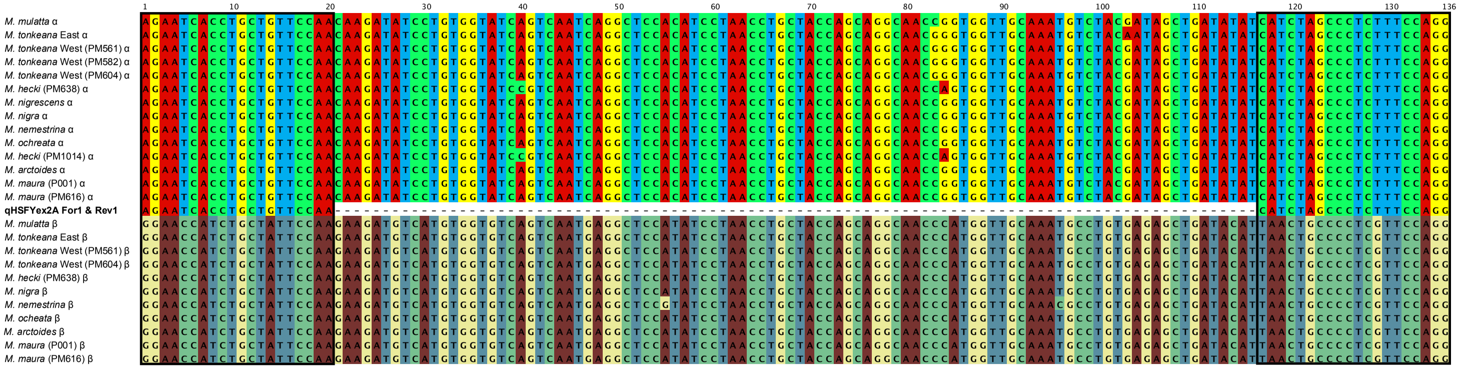

Figure S11: **Alignment of the qPCR amplicon sequence of *HSFY*-a and its primers for all of the macaque samples.** The sites corresponding to the forward and reverse primer sequences are enclosed in black boxes on the left and right, respectively. Washed-out sequences below the primers show *HSFY*-b sequences from selected macaques, which the primers are designed to *not* amplify.

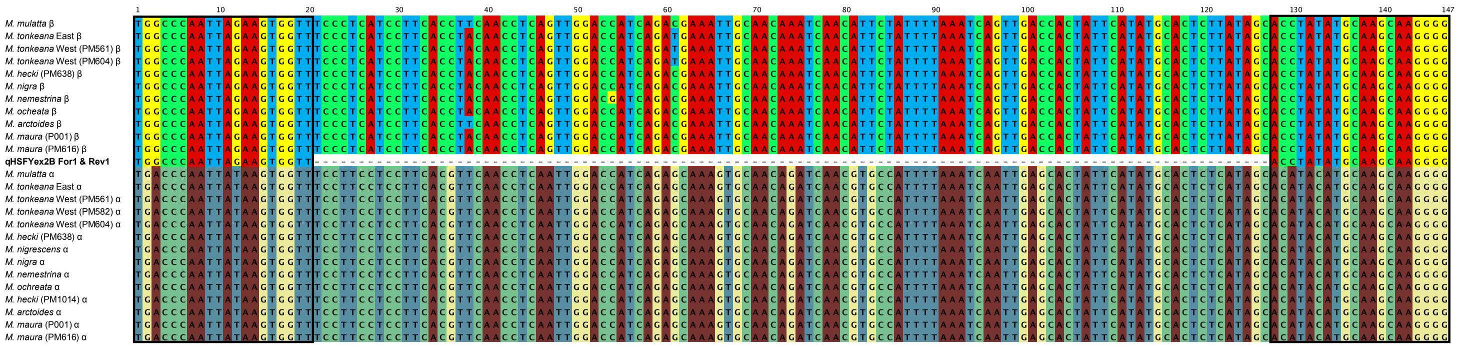

Figure S12: **Alignment of the qPCR amplicon sequence of *HSFY*-b and its primers for select macaque samples.** The sites corresponding to the forward and reverse primer sequences are enclosed in black boxes on the left and right, respectively. Washed-out sequences below the primers show *HSFY*-a sequences from macaques, which the primers are designed to *not* amplify.

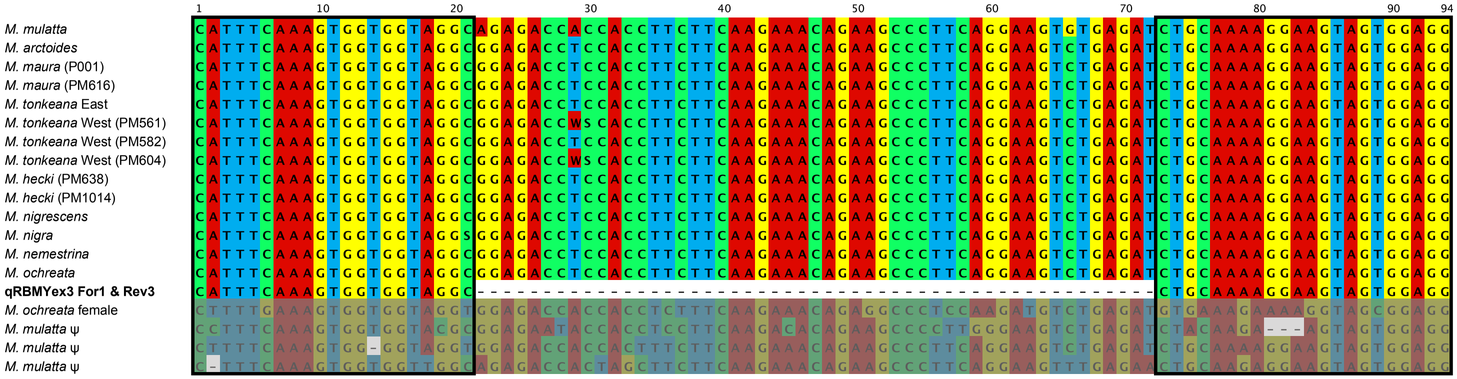

Figure S13: **Alignment of the qPCR amplicon sequence of *RBMY* and its primers for all of the macaque samples.** The sites corresponding to the forward and reverse primer sequences are enclosed in black boxes on the left and right, respectively. Greyed-out sequences below the primers show a highly similar sequence isolated from a *M. tonkeana* female (sample PF559; as well as macaque males) and *RBMY* pseudogene sequences from *M. mulatta*; primers were designed to avoid amplification of any of these non-target sequences.

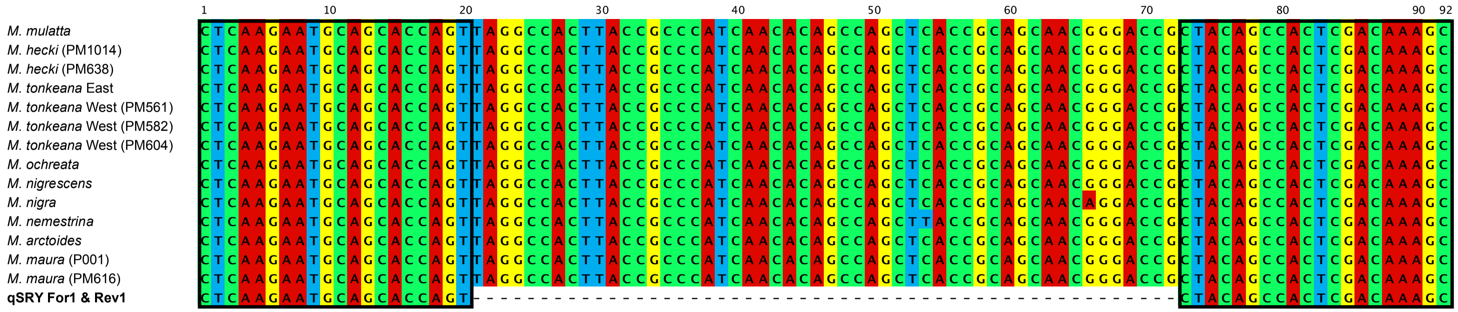

Figure S14: **Alignment of the qPCR amplicon sequence of *SRY* and its primers for all of the macaque samples.** The sites corresponding to the forward and reverse primer sequences are enclosed in black boxes on the left and right, respectively.

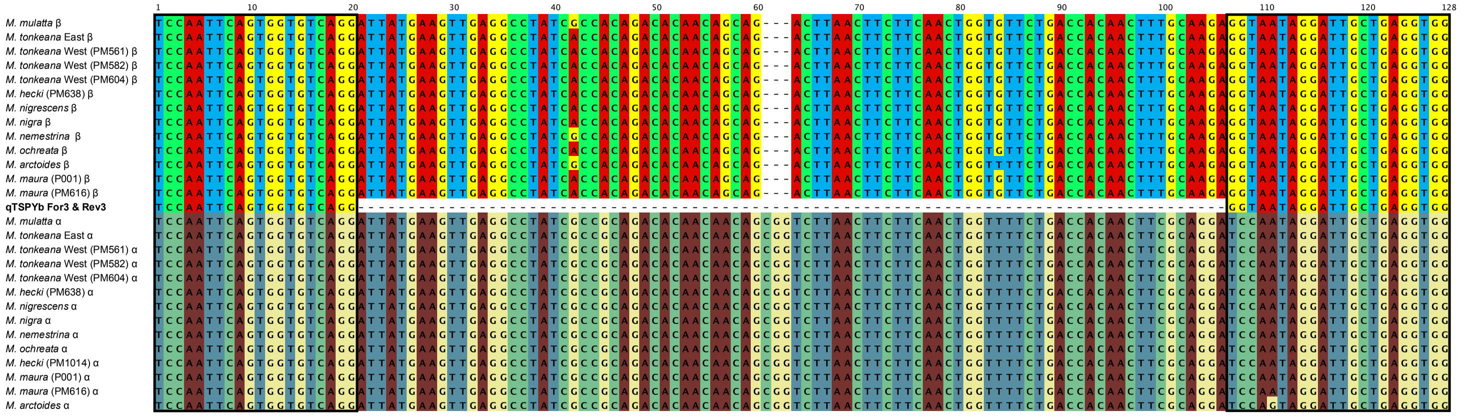

Figure S15: **Alignment of the qPCR amplicon sequence of *TSPY*-b and its primers for select macaque samples.** The sites corresponding to the forward and reverse primer sequences are enclosed in black boxes on the left and right, respectively. Washed-out sequences below the primers show *TSPY*-a sequences from macaques, which the primers are designed to *not* amplify.

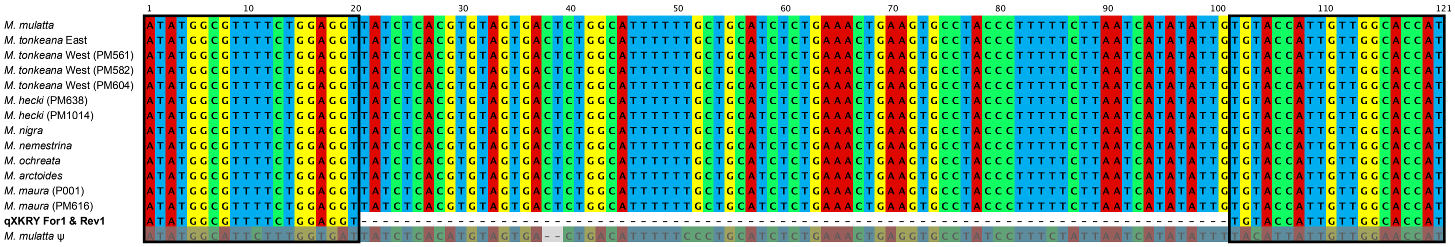

Figure S16: **Alignment of the qPCR amplicon sequence of *XKRY* and its primers for select macaque samples.** The sites corresponding to the forward and reverse primer sequences are enclosed in black boxes on the left and right, respectively. Greyed-out sequences below the primers show a *XKRY* pseudogene sequence from *M. mulatta*, which the primers are designed to *not* amplify.

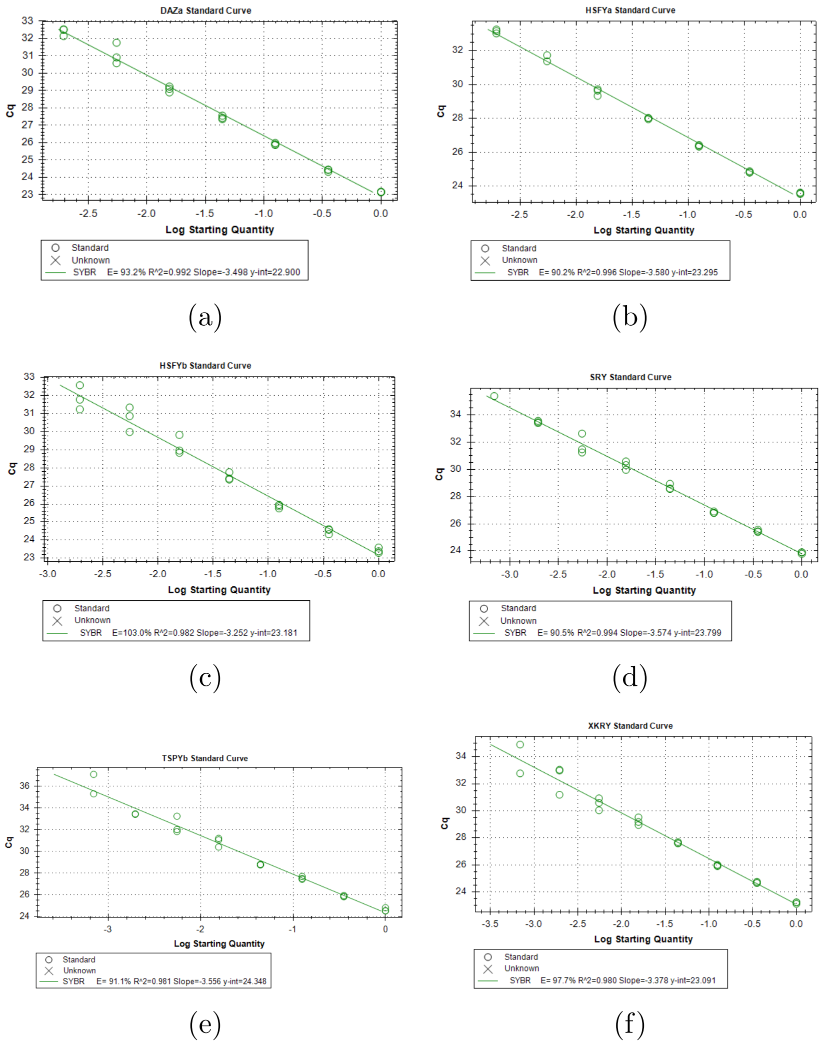

Figure S17: Standard curves for the set of preliminary assays performed using the iTaq Fast SYBR Green Supermix with ROX. (a) shows assay DAZa, (b) shows assay HSFYa, (c) shows assay HSFYb, (d) shows assay SRY, (e) shows assay TSPYb, and (f) shows assay XKRY.

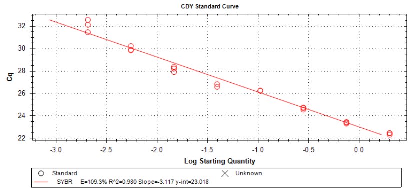

(a)

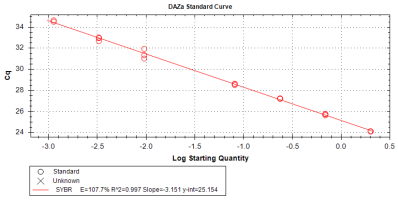

(b)

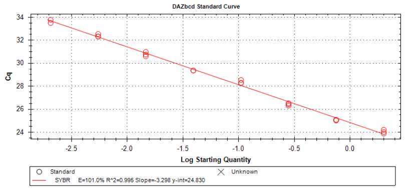

(c)

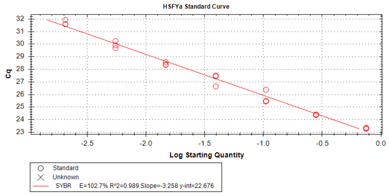

(d)

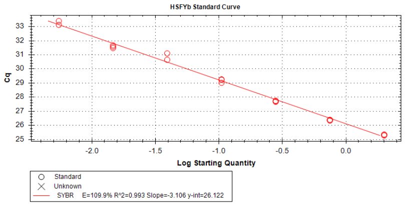

(e)

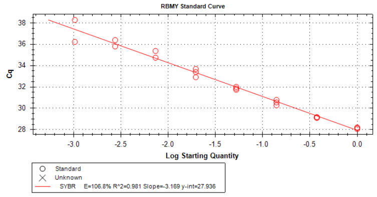

(f)

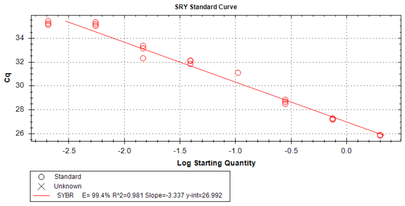

(g)

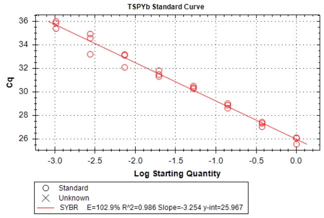

(h)

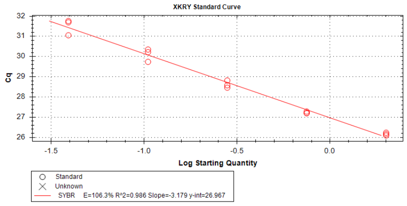

(i)

Figure S18: Standard curves for the assays performed using the SsoFast EvaGreen Supermix. (a) shows CDY, (b) shows DAZa, (c) shows DAZbcd, (d) shows HSFYa, (e) shows HSFYb, (f) shows RBMY, (g) shows SRY, (h) shows TSPYb, and (i) shows XKRY.

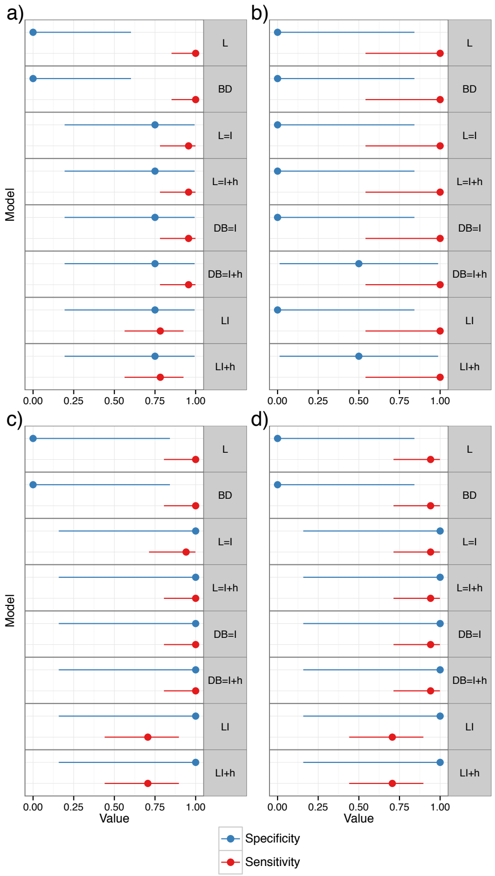

Figure S19: **The specificity and sensitivity of models in identifying the ancestral state of msrY-linked gene families.** The true negative rate (i.e. specificity) and the true positive rate (i.e. sensitivity) of each model to correctly identify gene families that were absent (i.e. true negatives) or present (i.e. true positives) in the MRCAs of Old World primates (a-c) and Hominini (d) as determined by Cortez et al. (2014); Hughes, Skaletsky, Brown, et al. (2012); Skaletsky et al. (2003). Points show the estimated values and lines show 95% confidence intervals estimated from a Binomial distribution with $p = 0.5$ and n equal to the number of gene families. **a)** Models are fit to the complete Y-chromosome data. There are 27 gene families, of which four are assumed to be absent in the MRCA: the two X-transposed gene families, *PCDH11Y* and *TGIF2LY*, and the AGs *PRY* and *VCY*. **b)** Models are fit to AG data. There are eight gene families, of which the same two AGs are absent as in a). **c)** Models are fit to the Y-linked singleton data. there are 19 gene families, of which the same two singletons are absent as in a). **d)** Models are fit as in c) but compared against the marginal ancestral reconstruction at the Hominini MRCA, where the same two singletons are absent as in a)
